# The Bacterial Community Structure of Hydrocarbon-Polluted Marine Environments as the Basis for the Definition of an Ecological Index of Hydrocarbon Exposure

**DOI:** 10.1264/jsme2.ME14028

**Published:** 2014-06-24

**Authors:** Mariana Lozada, Magalí S. Marcos, Marta G. Commendatore, Mónica N. Gil, Hebe M. Dionisi

**Affiliations:** 1 Laboratorio de Microbiología Ambiental, Centro Nacional Patagónico (CENPAT—CONICET), Blvd. Brown 2915, U9120ACD, Puerto Madryn, Chubut Province, Argentina; 2 Laboratorio de Oceanografía Química y Contaminación de Aguas, CENPAT—CONICET, Puerto Madryn, Chubut Province, Argentina

**Keywords:** hydrocarbon pollution, coastal marine environments, bacterial community structure, pyrosequencing, ecological indicators

## Abstract

The aim of this study was to design a molecular biological tool, using information provided by amplicon pyrosequencing of 16S rRNA genes, that could be suitable for environmental assessment and bioremediation in marine ecosystems. We selected 63 bacterial genera that were previously linked to hydrocarbon biodegradation, representing a minimum sample of the bacterial guild associated with this process. We defined an ecological indicator (ecological index of hydrocarbon exposure, EIHE) using the relative abundance values of these genera obtained by pyrotag analysis. This index reflects the proportion of the bacterial community that is potentially capable of biodegrading hydrocarbons. When the bacterial community structures of intertidal sediments from two sites with different pollution histories were analyzed, 16 of the selected genera (25%) were significantly overrepresented with respect to the pristine site, in at least one of the samples from the polluted site. Although the relative abundances of individual genera associated with hydrocarbon biodegradation were generally low in samples from the polluted site, EIHE values were 4 times higher than those in the pristine sample, with at least 5% of the bacterial community in the sediments being represented by the selected genera. EIHE values were also calculated in other oil-exposed marine sediments as well as in seawater using public datasets from experimental systems and field studies. In all cases, the EIHE was significantly higher in oiled than in unpolluted samples, suggesting that this tool could be used as an estimator of the hydrocarbon-degrading potential of microbial communities.

Hydrocarbon pollution is a widespread environmental concern in coastal ecosystems, with inputs being attributed to oil extraction and transportation activities, shipping, urban runoff, and pollutant discharges from industrial activities (12). Due to the hydrophobic nature of these compounds, sediments act as a major sink for hydrocarbons (30). As a consequence, coastal marine sediments can accumulate high concentrations of these contaminants, with potential detrimental consequences for ecosystem health, as well as provision of goods and services (34). Although bioremediation is considered to be a promising technology for reducing these risks, one of the greatest constraints for its widespread adoption is the difficulty in confidently predicting and subsequently meeting end-point levels (13). A series of advanced techniques, collectively called environmental molecular diagnostics, have recently been applied in this field to further analyze the biological and chemical characteristics of polluted sites (43). When used in conjunction with traditional analytical methods (*e.g.*, environmental parameters and pollutant concentrations), these tools can bring a new perspective to all stages in the decision-making process, *i.e.*, site characterization, remediation, monitoring, and closure (43). These multiple lines of evidence will lead to better-informed decisions that are fundamental for increasing the efficiency and decreasing the costs of bioremediation technologies (41). Some of these emerging tools are based on molecular biological techniques that use biomarker genes as targets. These tools can provide important microbiological evidence, such as whether key contaminant-degrading microorganisms are present at the site or if a biostimulation treatment was able to elicit the growth of pollutant-degrading microorganisms (43).

The sequencing of amplicons in 16S rRNA gene hypervariable regions using next-generation sequencing technologies is currently considered to be one of the best approaches for describing microbial communities (44). In contrast to other molecular methods such as PCR clone libraries, this technique avoids cloning biases and is able to reach high coverage values, which is essential for analyzing highly diverse microbial communities (49). Analyses based on 16S rRNA gene amplicon pyrosequencing have successfully been used to characterize bacterial communities in polluted marine environments (24), sediment microcosms treated with oil (10, 14, 18), and soil bioremediation experiments (1). Using this approach, it was possible to detect differences in specific bacterial genera following oil exposure (14, 18). We hypothesized that, although our understanding of hydrocarbon biodegradation processes remains incomplete, deep sequencing of 16S rRNA gene amplicons may be able to provide reliable information concerning community structures in polluted sites, which will be of value for estimating pollutant biodegradation potential. The aim of this study was to design an ecological indicator that could condense the taxonomic information provided by pyrotag analysis into a value indicative of the proportion of the bacterial community potentially capable of biodegrading hydrocarbons. This indicator was applied to a sequence dataset generated herein, which corresponded to coastal sediment samples obtained at two sites with different hydrocarbon exposure histories. We also evaluated this ecological indicator using other next-generation sequence datasets from marine environments.

## Materials and Methods

### Ecological index of hydrocarbon exposure

The ecological index of hydrocarbon exposure (EIHE) was defined as ∑*_i=1_^i=n^* *HCdeg*, where *HCdeg* are each of the relative abundances (as a percentage of the total reads) of *n* representative genera associated with hydrocarbon biodegradation. Sixty-three genera were selected from the literature (Table S1). To calculate this index, the relative abundance of each genus was extracted from the classification of pyrotag sequences performed with the software mothur v.1.29.1 (36). An R script (http://www.r-project.org/) was generated to automate this procedure (available in the Supplemental Material), which parses the mothur output table (the *.tax.summary* file, generated from the *classify.seqs* command), calculates percent abundances for the whole dataset, extracts the percent abundance of the selected genera, and calculates their sum total in each sample. The output is a space-delimited text file with the name of each sample and its corresponding index value. A table with the percent abundance of the selected genera is also generated as an output. Public sequence databases are sometimes already demultiplexed and barcode information is no longer available; therefore, an alternative script for the processing of multiple one-sample summary files is also available in Supplemental Material.

### Sampling and physicochemical analyses

Sediments (top 3 cm) were sampled along the low-tide line at ten random points using acrylic cores with an inner diameter of 4.4 cm, were then placed collectively in sterile glass flasks in order to generate a composite sample and stored at 4°C during transport to the laboratory. Each sample was mixed thoroughly and stored at −80°C for molecular analyses, or at −20°C for physical and chemical analyses. Sediment temperature, pH, and oxidoreductive potential (ORP) were measured *in situ* using a SG8 SevenGo pro™ pH field kit (Mettler-Toledo, Columbus, OH). Texture was measured by dry sieving (fine grains <63 μm, sand 63 μm to 2 mm, and gravel >2 mm). Organic matter content was estimated by weight loss after 4 h at 450°C. Total NH_4_^+^ was determined by extraction with 2 N KCl (22), centrifugation, and analysis of the supernatant. Analytical techniques for nutrients were carried out according to Strickland and Parsons (42).

Hydrocarbon content was analyzed by high-resolution gas chromatography according to Commendatore and collaborators (8, 9). The aliphatic fraction was subjected to high-resolution gas chromatography on a Thermo Trace gas chromatograph with a TriPlus Autosampler (Thermo Electron Corporation, Whaltman, MA, USA), equipped with a J & W DB5 fused silica column (30×0.25×25), split/splitless capillary injection system, and flame ionization detector (FID). Regarding *n*-alkanes from the *n*-C20 to *n*-C28 range, recovery assays of spiked pristine samples resulted in 95±12%. The percentage of relative deviation (RDP) for individual aliphatic hydrocarbons varied from 0.4 to 9%, and the detection limit (LOD) was between 5 and 10 ng g^−1^. The identification of resolved aliphatic hydrocarbons was achieved by comparing retention times with the corresponding standards (Chem Service, West Chester, PA, USA). Total resolved aliphatics (TRA) and unresolved complex mixture (UCM) were calculated using the mean response factors of *n*-alkanes. Individual *n*-alkane concentrations from *n*-C10 to *n*-C35, Pristane (Pr) and Phytane (Ph) isoprenoid levels, total resolved *n*-alkanes (∑*n*alk), TRA, UCM, and total aliphatic hydrocarbons (TAH=TRA+UCM) were calculated for each sample. The evaluation indices selected for this study were *n*-C17/Pristane (*n*C17/Pr) and *n*-C18/Phytane (*n*C18/Ph) in order to evaluate the relative biodegradation of *n*-alkanes (7, 8).

### Extraction of environmental DNA

High purity, high molecular mass DNA was purified in duplicate from 0.5 to 0.8 g wet weight sediment using the FastDNA SPIN kit for soil (MP Biomedicals, Santa Ana, CA), as previously described (26). Two extractions per sample were combined before further analysis. DNA concentrations were measured using Hoechst 33258 dye (Amersham Biosciences, Piscataway, NJ) in a Hoefer DyNA Quant 200 fluorometer (Hoefer Scientific Instruments, San Francisco, CA). The quality of the extracted DNA was evaluated by agarose gel electrophoresis and qPCR of the bacterial 16S rRNA gene (data not shown), as previously described (27).

### Pyrosequencing and bioinformatic analyses

Sediment samples were analyzed by 16S rRNA amplicon pyrosequencing with primers targeting the V4 hypervariable region. PCR amplification and pyrosequencing were performed at INDEAR, Argentina, as previously described (18). Sequences were analyzed with mothur following the standard operating procedure for next-generation sequence datasets as previously described (38), with modifications. Briefly, raw sequences were trimmed with mothur based on quality parameters (minimum sequence length of 200 bp, absence of ambiguous bases, 2 mismatches with the primers and 1 with the barcode, average quality value of 35 in a quality window size of 50). In OTU-based analyses, an alignment was performed with the aligned SILVA reference database as a template (32, 37). The alignment was filtered to conserve 85% of the sequences that overlapped in the same alignment space, and was then used to perform OTU clustering. Before clustering, sequences were pre-clustered at 99% to account for sequencing errors (20), and chimeras were removed by Chimera Slayer (19). OTU lists were calculated at a distance of 6% for the construction of rarefaction curves and diversity estimators. The alpha diversity metrics calculated included Chao1 (6), Good’s coverage index (17), and Shannon’s Diversity Index (39). Sequences were taxonomically classified with mothur using the SILVA reference database and RDP Bayesian classifier (45), up to the lowest possible rank, using a bootstrap value of 50% as the cut-off for reliable assignment. Public sequence datasets from other studies were obtained either from SRA (http://www.ncbi.nlm.nih.gov/sra) or GenBank (http://www.ncbi.nlm.nih.gov/genbank/), and were analyzed as described above.

### Statistical Analyses

Sequence datasets from studies with less than three replicates per condition/site were analyzed using the two-sample permutation test for differences in proportions (Fisher’s exact test with the Newcombe-Wilson method to calculate confidence intervals). When more than three replicates per condition were available, these were analyzed using the two-group Welch’s *t*-test (a variation of the Student’s *t*-test used when two groups cannot be assumed to have equal variance) or Kruskal-Wallis non-parametric median test with multiple comparisons. Analyses were performed both at the level of individual genera and at the hydrocarbon biodegradation guild level, at which the condition for guild membership (“hydrocarbon biodegradation guild” vs. “others”) was defined based on the list of the 63 genera linked to hydrocarbon biodegradation and selected in this study (Table S1). Analyses were performed with STAMP software (Statistical Analysis of Metagenomic Profiles, http://kiwi.cs.dal.ca/Software/STAMP).

### Sequence Accession Numbers

Partial 16S rRNA gene sequence datasets from this study are available at the SRA database of NCBI under accession number SRA049611 (samples SRS290218, SRS290219, and SRS290243).

## Results

### Definition of an ecological index of hydrocarbon exposure

Sixty-three genera were chosen from the literature, based on the report of at least one hydrocarbon-degrading strain and evidence of their role in hydrocarbon biodegradation in environmental samples (listed in Table S1). Most of these genera belonged to the phylum *Proteobacteria* (71.4%), followed by *Actinobacteria* (19%), *Bacteroidetes* (4.8%), and *Firmicutes* (4.8%), and included bacteria isolated from marine as well as terrestrial environments. The descriptive information provided by next-generation sequencing of the 16S rRNA gene amplicons was used to calculate a concise parameter that could express the overall abundance of these genera. This parameter, which we named “ecological index of hydrocarbon exposure” (EIHE), was calculated as the total sum of the relative abundances of these genera, expressed as a percentage of the total reads (for details see the Materials and Methods section). We generated scripts for the R software environment, in order to calculate this index in a semi-automated manner directly from the output of commonly used pyrotag analysis pipelines (see Appendix in Supplemental Material).

### Evaluation of the EIHE in intertidal sediments of Patagonia

Two distinct sites of the Patagonian South Atlantic coast were chosen to evaluate the EIHE. These sites have different histories of hydrocarbon pollution while belonging to the same marine ecoregion (“North Patagonian Gulfs”) (40): Fracasso Beach (PF) and Cordova Cove (CC) (Fig. S1). PF is located in the San José Gulf, in the Natural Protected Area of Valdes Peninsula. Site CC, on the other hand, is located in the San Jorge Gulf in an area that is chronically exposed to hydrocarbon pollution due to crude oil exploitation and transportation as well as fishing activities. Approximately 100 d before sampling, an oil spill covered more than 4 km of the CC site, thereby resulting in a fresh input of hydrocarbons. Two composite samples were retrieved at the CC site and one at the PF site (Table S2). As expected, samples obtained at the CC site contained high aliphatic and polycyclic aromatic hydrocarbon (PAH) concentrations, while t he sample obtained at the PF site showed negligible hydrocarbon concentrations (Table S2). Biodegradation diagnostic indices based on the ratio between biodegradable and recalcitrant hydrocarbons (*n*-C17/Pristane and *n*-C18/Phytane) (7, 8) were calculated in order to evaluate the relative biodegradation state of the oil mixtures in the samples. Values in sediments from the CC site ranged from 0.3–1.1 (Table S2). Values of 1 or less in these indices suggested the presence of degraded oil, and this was associated with the disappearance of the most readily biodegradable hydrocarbons (7). These results indicated that bacterial communities were actively taking part in the biodegradation of hydrocarbons at this polluted site.

Pyrosequencing of 16S rRNA gene amplicons resulted in a total of 105,942 reads. After discarding low-quality sequences, chimeras and reads assigned to chloroplasts, more than 20,000 reads per sample were analyzed in further steps. Richness estimators such as Chao1 revealed the high diversity of OTUs, with coverage values above 90% in all analyzed samples (Table 1). If we assumed that 6% was a conservative estimation of the genetic distance between OTUs at the genus level (35), and Chao1 as an estimator of the minimum richness expected in a sample (6), we estimated that there were at least 3,000 genera inhabiting these intertidal sediments. This bacterial diversity was similar for the PF08 and CC08-2 samples (Shannon’s Diversity index >6), while CC08-1 showed a lower diversity value (Shannon’s diversity index of 5, Table 1). Low dominance was observed, with the most abundant OTU in the datasets being represented by 2 to 12% of the reads (Table 1).

**Fig. 1:**
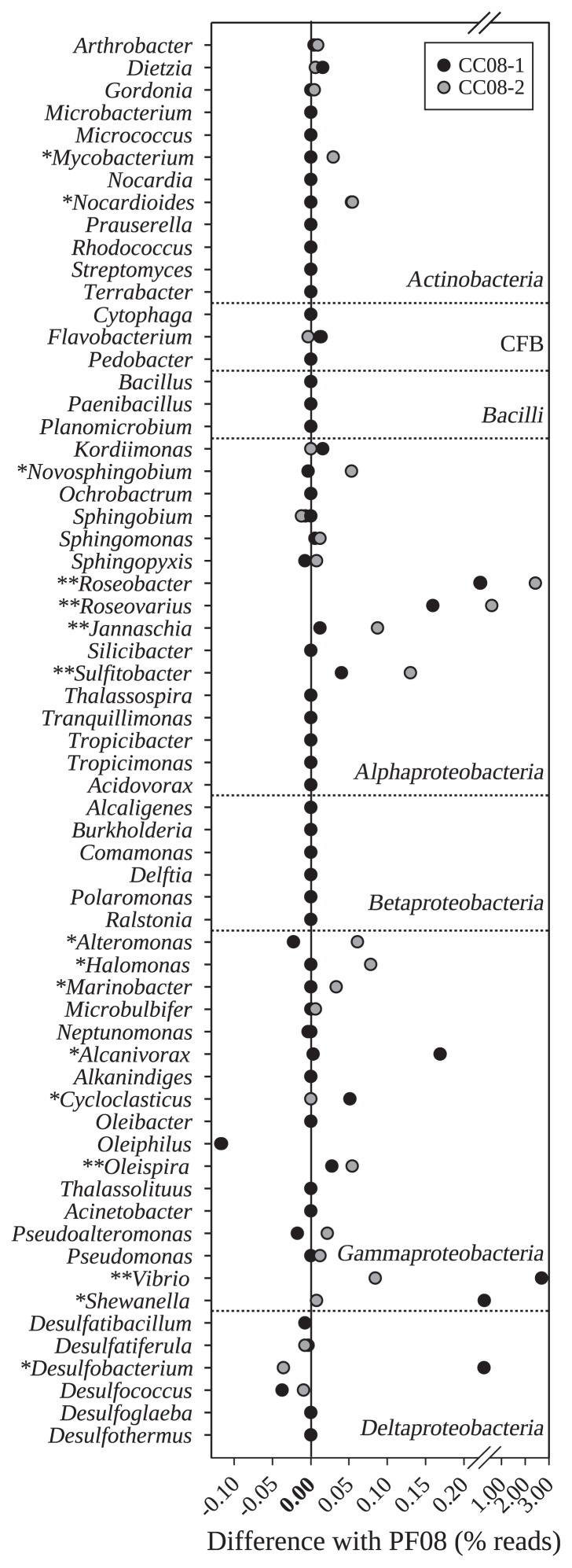
Differences in the proportions of reads assigned to genera linked to hydrocarbon biodegradation, in samples from the CC site in relation to unpolluted sample PF08. The values were calculated for each genus and each polluted sample as percentage reads in the polluted sample (CC08-1 or CC08-2) minus the percentage reads in PF08. Zero values corresponded to equal relative abundances (no differences between polluted and unpolluted samples). Genera with significant differences in their proportions with respect to the unpolluted sample were marked with asterisks (one or two asterisks for abundances differing significantly in one or both polluted samples, respectively). The results of the statistical analysis are shown in detail in Fig. S2. The complete list of the genera with their corresponding references is available in Table S1.

We analyzed the sequence datasets generated from samples collected at the CC and PF sites with a focus on the 63 selected bacterial genera previously linked to hydrocarbon biodegradation. Many bacterial genera known to play a role in hydrocarbon biodegradation were present at higher abundances in polluted samples CC08-1 and CC08-2 than in the pristine sample PF08 (departures from zero, Fig. 1). Sixteen genera (25%) showed significantly higher abundances in at least one of the polluted samples than in the pristine sample. Moreover, 6 of the 63 selected genera were significantly more abundant in both polluted samples. These were *Roseobacter*, *Roseovarius*, *Jannaschia*, *Sulfitobacter*, *Vibrio*, and *Oleispira*. All but one (*Sulfitobacter*) of these genera were reported to have strains isolated from marine environments (Table S1). Only one genus (*Oleiphilus*) was in significantly higher abundance in PF08, but absent in both samples from the polluted site. Overall, these results suggested the highly specific adaptation of the sediment bacterial community at this site as a result of exposure to these pollutants, which was consistently detected as an excess in the abundance of reads assigned to specific genera.

Although various bacterial genera linked to hydrocarbon biodegradation were present in higher abundances in the polluted samples than in the pristine sample, the relative abundance of each individual genus was generally small. However, differences between polluted and pristine samples were more marked when the relative abundances of all 63 selected genera were combined. We used a two-sample permutation test for differences in proportions to analyze if differences in EIHE values could be considered significant (Fig. S2, see the Materials and Methods section for details of the statistical analysis). On the left of Fig. S2, the sum of the proportions of the genera accounting for the index (the EIHE value) is shown as a percentage of the total reads for each of the two samples being compared. On the other hand, the difference between these two proportions (filled circle), the corresponding 95% confidence intervals (error bar) and *p*-values, are shown on the right of Fig. S2. Fig. S2A shows the results obtained when polluted samples CC08-1 and CC08-2 were compared with pristine sample PF08. The EIHE index values in the polluted samples were 5.2 and 4.8, and were significantly higher than that in the pristine sample, the value of which was 1 (*p*<10^−15^, Fig. S2A).

### Evaluation of the EIHE in datasets from previously published studies

The same analysis was applied to five previously published datasets involving sediments and seawater, in both experimental systems and environmental field assessments. Overall, these studies included an analysis of 76 samples and more than 350,000 reads. The information for these datasets has been summarized in Table 2. The results of the statistical analysis are shown in Fig. S2B to S2F. The EIHE was indicative in all cases of the hydrocarbon exposure status of the samples. For example, chronically polluted Subantarctic sediments exposed to crude oil for 20 d in an experimental system (18) had an EIHE value of 16.5, while in the control treatment, the index was less than half this value (Fig. S2B). The EIHE value markedly increased when nutrients were added in addition to crude oil, with more than 80% of the reads being assigned to the selected genera (Fig. S2B). On the other hand, a rapid increase was observed in the EIHE values with respect to controls in temperate coastal mudflat sediments exposed to crude oil, and this was followed by a decrease after 21 d (Fig. S2C). These changes were consistent with previous findings in which the response of the sediment bacterial community was only evident in the first stages of the experiment (10). Mangrove sediments microcosms (14) behaved in a similar manner, and, more importantly, EIHE values were proportional to the amount of oil added in the experiment (Fig. S2D). The index was able to clearly differentiate between oiled and unoiled samples from a field study involving beach sediments impacted by the Deepwater Horizon spill (Fig. S2E) (24). A trend was also observed when samples from two different sampling events that occurred 30 and 60 d after the spill were analyzed separately (PB1 and PB2 respectively, Fig. S2G). Moreover, very low EIHE values were observed at a non-impacted site (SGI, Fig. S2G). Seawater naphthalene biodegradation experiments also showed marked increases in EIHE values in samples exposed to oil (Fig. S2F).

The relative contribution of the various genera composing the index varied in the different studies (Fig. S3). For example, in the Subantarctic sediments dataset (Fig. S3B), *Nocardioides*, *Sphingopyxis* and members of the *Roseobacter* clade mostly accounted for the sediment EIHE value. However, when nutrients were amended, in addition to oil, *Alcanivorax* and *Thalassospira* were the dominant genera that accounted for the EIHE value. In temperate coastal mudflat sediments, *Alcanivorax* and *Cycloclasticus* were the main genera that increased after oil exposure (Fig. S3C). In the case of tropical mangrove sediments microcosms, the EIHE values in oiled microcosms were largely attributed to *Marinobacter* (Fig. S3D), while *Alcanivorax* was the main genus accounting for the increase in index values in oiled samples from the Pensacola Beach dataset, followed by *Marinobacter* (Fig. S3E). On the other hand, seawater microcosms exposed to naphthalene showed a different response depending on the microbial community of origin and the temperature of incubation (Fig. S3F). In the case of Arctic seawater, genera *Neptunomonas*, *Pseudoalteromonas*, and *Oleispira* increased their relative abundance when incubated at 15°C, while only *Pseudoalteromonas* strongly increased when the experimental systems were exposed to low temperatures (0.5, 4 and 8°C). In contrast, in temperate seawater communities, *Cycloclasticus* was the main genus responsible for the increase of EIHE values at all temperatures tested when a response was observed.

## Discussion

In the present study, we used information obtained from sequencing 16S rRNA gene fragments amplified from environmental samples to define an ecological indicator, which we called “ecological index of hydrocarbon exposure” (EIHE). The purpose of this index was to summarize information concerning the overall abundance of potential hydrocarbon-degrading bacterial genera in an environmental sample into a single parameter. Due to the usually low abundance of the individually targeted genera in highly diverse microbial communities, the calculation of this index requires the use of high-throughput sequencing platforms such as 454/Roche. Pyrosequencing of 16S rRNA gene amplicons, although not free of biases, has proved to be a robust and reproducible technique for analyzing microbial community structures, and is capable of reliably recovering template abundances in a semi-quantitative manner (31). The Illumina platform has significantly increased sequencing depth and reduced per base costs, and is now being used to analyze microbial community structures (15). Rapid advances in sequencing technologies could facilitate the application of the ecological index defined in this study to environmental diagnostics.

Molecular biological tools target biomolecules that provide information about organisms and processes relevant to contaminant assessments and/or remediation (41). Specific DNA sequences are most commonly targeted by these assays, and belong to either functional or phylogenetic biomarker genes. The former presents the advantage of providing direct information concerning the potential of the microbial community to degrade the pollutants. However, there is still limited knowledge concerning functional biomarker genes (43), in particular those linked to hydrocarbon biodegradation (18, 27). The second constraint for the use of functional biomarker genes specific to hydrocarbon biodegradation is their high diversity (4, 5, 18, 21), which hinders the design of high-coverage molecular assays. The phylogenetic approach used in this study overcomes some of these problems, by allowing the detection of a broad range of microorganisms that could be impossible to cover with a single molecular assay based on a functional biomarker gene. In addition, phylogenetic biomarkers can detect genera for which catabolic gene information is not yet available. Of the 63 genera chosen to design the EIHE, there were 17 for which the biodegradation genes have not yet been described. For example, members of the genus *Oleispira* were detected in Subantarctic sediment samples and Arctic seawater. Members of this genus are psychrophilic obligate hydrocarbonoclastic marine bacteria that were initially detected in cold or high latitude regions (*Oleispira antarctica*) (48). However, a mesophilic species from this genus has recently been proposed (*Oleispira lenta*) (46) with similar characteristics regarding aliphatic hydrocarbon preferences, its genome has only recently been sequenced, and biodegradation genes have been annotated for one strain of this species (GenBank accession no. FO203512). The information provided by the 16S rRNA gene approach was, in this case, fundamental for detecting the contribution of these microorganisms to the potential hydrocarbon-degrading guild.

An agreement was observed between the proportion of pyrosequencing reads assigned to the *Cycloclasticus* genus in the samples reported in the present study and the relative abundances of the *phnA1* gene (a functional biomarker specific for this genus) previously estimated by qPCR in the same samples (27). According to pyrotag data, 0.05% of the sequences were assigned to the *Cycloclasticus* genus in CC08-1 (the more heavily polluted sample), while no sequences assigned to this genus could be detected in the pristine (PF08) and moderately polluted (CC08-2) samples. Using qPCR analysis, 10^5^ copies of the *phnA1* gene per μg of sediment DNA were estimated to be present in CC08-1, approximately 0.12% of the bacterial 16S rRNA gene abundance using the same technique (27). In the other two samples, the *phnA1* gene was either detected below the quantification limit (CC08-2) or could not be detected by qPCR analysis (PF08) (27). Other studies have shown similar agreements between pyrosequencing and qPCR data (*e.g.*, for *Alcanivorax*) (24), which suggests that although not exempt of biases, 16S rRNA gene amplicon pyrosequencing can provide at least a semiquantitative view of the bacterial community structure in environmental samples.

Although the bacterial populations detected by the EIHE may play a role in natural attenuation processes, the phylogenetic approach used in the present study was unable to provide information concerning the actual function of these bacterial populations in the sediments. For example, it is possible that some of the populations belonging to these genera do not have genetic information for the catabolic pathways, or that this genetic information is not being expressed. On the other hand, there could be yet unidentified hydrocarbon-degrading bacterial genera at this site that have been missed because there is still much to learn concerning hydrocarbon-degrading bacterial populations from marine environments (23, 29). For example, in one of the polluted sediment samples analyzed in this study (CC08-1), 15% of the reads were assigned to the *Psychromonas* genus. Although this genus has been identified in microcosms and field biodegradation experiments as potentially being involved in hydrocarbon biodegradation processes (3, 33), it was not included in the calculation of the index because no strains with hydrocarbon biodegradation abilities have yet been described. In addition, although deep sequencing of 16S rRNA gene amplicons is currently the most accurate and in-depth method available to study bacterial community structure, it still shows biases as well as incomplete coverage. For example, we found in the samples reported in this study that members of the *Flavobacteria* class were poorly represented (1 to 3.5% of the pyrotag reads, results not shown). The Bacteroidetes group (formerly *Cytophaga*–*Flavobacteria*–*Bacteroides*) is known to be well represented in coastal sediments and in general in the marine environment, in which they play a major role in the cycling of organic carbon (28, 49). This discrepancy was most probably due to poor amplification of this taxon with the V4 primers.

In environmental assessment studies, different types of biological indicators have been used to identify chemical stressors and their potential risk for ecosystem processes based on different levels of biological organization (16). Ecological indicators in particular use ecosystem parameters such as species diversity or population dynamics as a measure of ecological effects. In the present study, we defined the ecological indicator based on the relative abundance of the bacterial guild formed by potential hydrocarbon-degrading bacteria. As hydrocarbon biodegradation is a complex process that involves the breakdown of various molecular structures by various microbial populations, each of these structures could be used by more than one population (11). Therefore, some degree of functional redundancy is expected in the bacterial community. As a consequence, the specific genera that are enriched in each polluted sample may vary, as was observed in the polluted environmental samples used in this study that were obtained only meters apart and on the same day. Because the EIHE targets multiple microorganisms, it has the advantage of being able to distinguish between polluted and unpolluted samples regardless of differences in individual genera. This is the case for the different exposure conditions associated with each study, involving sediments from different origins (tropical mangrove sediments, intertidal sediments from temperate sites, and Subantarctic environments) and also seawater. The scripts developed for this purpose allow not only EIHE values to be obtained, but also provide information concerning the specific genera that are contributing to the index, both of which can be further analyzed with standard statistical methods.

In order to account for the contribution of terrestrial microorganisms to the coastal community, and also to be able to apply this index to other environments, we did not limit the selection to marine microorganisms. However, we only tested the EIHE in samples of marine origin. Similarly, only environments in aerobic conditions were evaluated (intertidal sediment samples, aerated slurries, and seawater microcosms). Therefore, although we included genera known for their anaerobic hydrocarbon biodegradation capabilities, the limited information available concerning this important process (47) can preclude obtaining a meaningful assessment of anaerobic environments. As more information concerning hydrocarbon-degrading bacteria is gained, new genera can be added for the calculation of this index, thereby improving its assessment ability. Alternatively, similar indices can be built based on the information available for each particular environment.

The determination of EIHE values has the potential to rapidly and reproducibly obtain microbiological evidence of the presence and relative abundance of genera potentially related to hydrocarbon biodegradation. It is a relatively simple technique that could precede or complement other molecular methods such as qPCR of functional biomarker genes, which require detailed knowledge about key biomarkers specific for the analyzed environment. Furthermore, this tool can be applied in a semi-automated high-throughput manner. However, further studies are needed to validate this molecular biological tool in the field, involving standardized procedures at all steps, including experimental design, sampling, laboratory procedures, and data analysis (25). The sampling design should take into account the analysis of multiple samples in order to account for environmental variability at the site, and ideally should include reference, non-polluted samples of the same site. It is also important to take into account that this approach, like all DNA-based molecular biological tools, can detect inactive or dead cells or even extracellular DNA. Therefore, the implementation of this index using RNA instead of DNA should overcome this problem to ultimately provide a more direct estimation of the active members in the hydrocarbon-degrading guild.

## Supplemental Materials



## Figures and Tables

**Table 1:** Ecological estimators calculated for the intertidal sediment samples PF08, CC08-1 and CC08-2

Parameter*	Sample		

	PF08	CC08-1	CC08-2
Read	26,357	25,450	33,196
%Cov	95	96	93
S_obs_	2,850	2,151	3,630
Chao1	4,371 (4,161–4,615)	3,589 (3,369–3,849)	6,084 (5,782–6,428)
% most abundant OTU	5.3	12	2.7
H	6.22 (6.20–6.25)	5.01 (4.98–5.04)	6.83 (6.81–6.85)

* Read: final number of reads obtained after removing primers, short (less than 200 bp) and low-quality sequences, chimeras and sequences assigned to chloroplasts; %Cov: coverage; S_obs_: observed OTUs; Chao1: Chao1 richness estimator; H: Shannon’s diversity index. Chao1 and H values were calculated using OTUs defined at 6% distance threshold, and a cut-off of 25,000 sequences. Lower and higher limits of 95% confidence intervals for Chao1 and H indices are shown between parentheses.

**Table 2:** Summarized information of the 16S rRNA gene amplicon datasets used in this study. More information is available in the Supplemental Material.

ID	General	System*	Ref	Accession	Region	*n*§	*N*†
0	Marine sediments from North Patagonia	field	This study	see the Material and Methods section	V4	3	85,003
1	Chronically polluted Subantarctic sediments	exp	(18)	SRA049611	V4	4	44,380
2	Coastal mudflat sediments	exp	(10)	FR865969–FR869630	V3	4	3,138
3	Mangrove sediments	exp	(14)	HM602044–HQ462469	V4	8	19,867
4	Beach sands impacted by the Deepwater Horizon oil spill	field	(24)	ERP000807	V1–V3	26	180,408
5	Seawater microcosms	exp	(2)	SRA061588	V3–V5	10	59,651

* exp: experimental system

§ Number of analyzed samples

† Total number of sequences used to calculate the EIHE (after trimming and discarding low quality sequences)
