# Supplementary material for: The Bacterial Community Structure of Hydrocarbon-Polluted Marine Environments as the Basis for the Definition of an Ecological Index of Hydrocarbon Exposure
Source: Microbes Environ. 2014 Jun 24;29(3):269–76. doi: 10.1264/jsme2.ME14028 (PMC4159038; doi:10.1264/jsme2.ME14028)
Supplement: Supplementary file 1 [file 29_269_s1.pdf]

Supplemental Materials

Tables

Table S1. Bacterial genera selected from the literature as reported to include hydrocarbon-degrading strains.

Phylum	Class	Genus	Gene info	Hydrocarbons	Ref
Actinobacteria	***Actinobacteria***	***Arthrobacter (FA)***	***phtAa***	***Monoaromatic compounds,* LMW-PAHs, monohaloalkanes**	**(45)**
Actinobacteria	*Actinobacteria*	*Dietzia*	*alkB*	*Medium and long chain alkanes,* monoaromatic compounds, PAHs	(59)
Actinobacteria	*Actinobacteria*	*Gordonia*	*ladA, alkB2, narAa*	*Short and long chain alkanes, LMW-PAHs*	(55)
Actinobacteria	*Actinobacteria*	*Microbacterium (FA)*	*alkB*	LMW and HMW-PAHs, crude oil	(47)
Actinobacteria	*Actinobacteria*	*Micrococcus (A)*	-	Monoaromatic compounds, LMW-PAHs, medium chain alkanes, crude oil	(29)
Actinobacteria	*Actinobacteria*	*Mycobacterium*	*phtAa, nidA, nidA3, bphA, alkB*	*Monoaromatic compounds, LMW and HMW-PAHs, short to long chain alkanes*	(54)
Actinobacteria	*Actinobacteria*	*Nocardia*	*alkB1, alkB2, alkB3, alkB4*	*Long chain alkanes,* LMW-PAHs	(42)
Actinobacteria	***Actinobacteria***	***Nocardioides***	***phdA, alkB***	***LMW-PAHs, short, medium and long chain alkanes***	**(20)**
Actinobacteria	*Actinobacteria*	*Prauserella*	*alkB*	*Short to long chain alkanes*	(48)
Actinobacteria	*Actinobacteria*	*Rhodococcus*	*alkB, alkB1* to *alkB7, bnzA1, bphA1, padAa2, narAa,*	Haloalkanes, *medium and long chain alkanes, monoaromatic compounds, LMW-PAHs* and HMW-PAHs	(41)

Phylum	Class	Genus	Gene info	Hydrocarbons	Ref
Actinobacteria	*Actinobacteria*	*Streptomyces*	*nidA* *alkB*	LMW and HMW-PAHs	(2)
Actinobacteria	*Actinobacteria*	*Terrabacter*	*phtA1, dbfA1*	*Monoaromatic compounds* and *LMW-PAHs*	(28)
Bacteroidetes	*Cytophagia*	*Cytophaga (FA)*	-	Crude oil	(33)
Bacteroidetes	*Flavobacteriia*	*Flavobacterium (FA)*	*alkB*	HMW-PAHs, diesel oil, crude oil	(50)
Bacteroidetes	*Sphingobacteriia*	*Pedobacter*	*alkB1* to *alkB7*	Monoaromatic compounds, LMW and HMW-PAHs, medium chain alkanes	(40)
Firmicutes	*Bacilli*	*Bacillus (FA)*	*alkB, alkB3, bphA1*	*Monoaromatic compounds*, LMW and HMW-PAHs, *medium and long chain alkanes*	(14)
Firmicutes	*Bacilli*	*Paenibacillus (FA, A)*	*bphA, dbfA1*	LMW-PAHs	(41)
Firmicutes	*Bacilli*	*Planomicrobium*	-	Alkanes, diesel oil	(13)
Proteobacteria	***Alphaproteobacteria***	***Kordiimonas***	***alkB, carAa***	**LMW and HMW-PAHs**	**(38)**
Proteobacteria	***Alphaproteobacteria***	***Novosphingobium***	***pheA1a, bphA1a, xylX***	***Monoaromatic compounds, LMW-PAHs* and HMW-PAHs**	**(49)**
Proteobacteria	*Alphaproteobacteria*	*Ochrobactrum*	*alkB*	LMW and HMW-PAHs, crude oil	(46)
Proteobacteria	*Alphaproteobacteria*	*Sphingobium*	*xylM, xylX, bphA1e, bphA1f*	*Monoaromatic compounds*, LMW and HMW-PAHs	(32)
Proteobacteria	***Alphaproteobacteria***	***Sphingomonas***	***carAa, dxnA1, ahdA1a, xylX, phnA1f***	**Monoaromatic compounds, *LMW and HMW-PAHs***	**(32)**
Proteobacteria	*Alphaproteobacteria*	*Sphingopyxis*	*thnA1*	*Monoaromatic compounds*, LMW and HMW-PAHs	(32)
Proteobacteria	***Alphaproteobacteria***	***Roseobacter***	***alkB***	**Crude oil**	**(37)**

Phylum	Class	Genus	Gene info	Hydrocarbons	Ref
			putative RHO		
Proteobacteria	***Alphaproteobacteria***	***Roseovarius***	**-**	**LMW and HMW-PAHs**	**(37)**
Proteobacteria	***Alphaproteobacteria***	***Jannaschia***	***alkB, P450, putative RHO***	**Alkanes, PAHs**	**(37)**
Proteobacteria	*Alphaproteobacteria*	*Silicibacter*	*alkB*	Dimethylsulfonio propionate	(37)
Proteobacteria	*Alphaproteobacteria*	*Sulfitobacter*	*alkB*	Crude oil	(5)
Proteobacteria	***Alphaproteobacteria***	***Thalassospira (FA)***	*alkB, putative phenylpropionate dioxygenase*	**LMW-PAHs**	**(34)**
Proteobacteria	***Alphaproteobacteria***	***Tranquillimonas***	**-**	**Medium to long chain alkanes**	**(22)**
Proteobacteria	***Alphaproteobacteria***	***Tropicibacter***	**-**	**Monoaromatic compounds, LMW-PAHs**	**(23)**
Proteobacteria	***Alphaproteobacteria***	***Tropicimonas (FA)***	**-**	**Medium to long chain alkanes**	**(24)**
Proteobacteria	*Betaproteobacteria*	*Acidovorax (FA)*	*alkB*	LMW-PAHs	(41)
Proteobacteria	*Betaproteobacteria*	*Alcaligenes (FA)*	*phnAC*	*LMW-PAHs*, HMW-PAHs, long chain alkanes	(52)
Proteobacteria	*Betaproteobacteria*	*Burkholderia*	*phnAC, alkB*	*LMW-PAHs*, HMW-PAHs, *medium to long chain alkanes*	(44)
Proteobacteria	*Betaproteobacteria*	*Comamonas (FA)*	*ndoA, nahAc, hcaE*	*LMW-PAHs*	(41)
Proteobacteria	*Betaproteobacteria*	*Delftia*	-	Monoaromatic compounds, LMW-PAHs	(43)
Proteobacteria	*Betaproteobacteria*	*Polaromonas*	*bphA1, nadC2, ndoA, nagAc, xylX*	*LMW-PAHs*, medium chain alkanes	(30)
Proteobacteria	*Betaproteobacteria*	*Ralstonia*	*amo,*	*Monoaromatic*	(44)

Phylum	Class	Genus	Gene info	Hydrocarbons	Ref
			alkB, ndoA,nag Ac xylX	*compounds, LMW-PAHs*	
Proteobacteria	***Gammaproteobacteria***	***Alteromonas***	***amo, ndoA***	**LMW and HMW-PAHs**	**(31)**
Proteobacteria	***Gammaproteobacteria***	***Halomonas (FA)***	**-**	**Monoaromatic compounds, LMW-PAHs, long chain alkanes, crude oil**	**(53)**
Proteobacteria	***Gammaproteobacteria***	***Marinobacter (FA)***	***AlkB1, alkM, benA, nahAc***	***Long chain alkanes, LMW-PAHs***	**(16)**
Proteobacteria	*Gammaproteobacteria*	*Microbulbifer*	-	Monoaromatic compounds, crude oil	(4)
Proteobacteria	***Gammaproteobacteria***	***Neptunomonas (FA)***	***nahAc***	***LMW-PAHs***	**(25)**
Proteobacteria	***Gammaproteobacteria***	***Alcanivorax (FA)***	***alkB1, alkB2, P450***	***Medium to long chain alkanes***	**(58)**
Proteobacteria	*Gammaproteobacteria*	*Alkanindiges*	-	Short to long chain alkanes	(3)
Proteobacteria	***Gammaproteobacteria***	***Cycloclasticus***	***bphA1, xylX, phnA1***	***Monoaromatic compounds, LMW-PAHs* and HMW-PAHs**	**(12)**
Proteobacteria	***Gammaproteobacteria***	***Oleibacter***	**-**	**Long chain alkanes**	**(51)**
Proteobacteria	***Gammaproteobacteria***	***Oleiphilus***	***alkB***	***Medium to long chain alkanes***	**(18)**
Proteobacteria	***Gammaproteobacteria***	***Oleispira (FA)***	***alkB***	***Alkanes***	**(57)**
Proteobacteria	***Gammaproteobacteria***	***Thalassolituus***	***alkB***	***Medium to long chain alkanes***	**(56)**
Proteobacteria	***Gammaproteobacteria***	***Acinetobacter***	***atdA, BenA, antA, alkB, alkMa, alkMb***	***Short to long chain alkanes, monoaromatic compounds,* LMW-PAHs**	**(6)**
Proteobacteria	***Gammaproteobacteria***	***Pseudoalteromonas (FA)***	***xylX, tmlW***	**Monoaromatic compounds, LMW-PAHs**	**(26)**
Proteobacteria	***Gammaproteobacteria***	***Pseudomonas (FA)***	***nahAc, ndoB, doxB,***	***Monoaromatic compounds, LMW-PAHs* and**	**(15)**

Phylum	Class	Genus	Gene info	Hydrocarbons	Ref
			pahA3 pahAc, bphA1, ditA1, carAa, tcbAa, xylX, alkB, alkB1, alkB2	**HMW-PAHs, *short to long chain alkanes***	
Proteobacteria	***Gammaproteobacteria***	***Vibrio (FA)***	***alkB, hcaE***	**LMW-PAHs**	**(27)**
Proteobacteria	***Gammaproteobacteria***	***Shewanella (FA)***	***alkB, hcaE***	**Crude oil**	**(17)**
Proteobacteria	*Deltaproteobacteria*	*Desulfatibacillum (A)*	-	Long chain alkanes, médium to long chain alkenes	(9)
Proteobacteria	*Deltaproteobacteria*	*Desulfatiferula (A)*	-	Long chain alkenes	(8)
Proteobacteria	*Deltaproteobacteria*	*Desulfobacterium (A)*	-	Monoaromatic compounds, LMW-PAHs, long chain alkanes	(21)
Proteobacteria	*Deltaproteobacteria*	*Desulfococcus (A)*	-	Long chain alkanes	(1)
Proteobacteria	*Deltaproteobacteria*	*Desulfoglaeba (A)*	-	Medium chain alkanes	(10)
Proteobacteria	*Deltaproteobacteria*	*Desulfothermus (A)*	-	Medium to long chain alkanes	(36)

Boldtype: genera including marine hydrocarbon degrading strains. Genera including anaerobic or facultatively anaerobic strains are identified as *(A)* or *(FA)*, respectively, next to the genus name. **Ref:** references. Hydrocarbons for which degrading enzymes are known are shown in italics.

Table S2. Sampling locations, dates and parameters measured in the sediments used for this study

Site	Sample	Lat; Long	Date	Temp (°C)	pH	ORP (mV)	Org mat (%)	Total NH_4^+ (nmol/g dws)	Granulometry(%)			AliHC (µg/g dws)	Biod. index	PAHs (µg/kg dws) (39)
									Gravel	Sand	Fine			
Fracasso Beach (PF)	PF08	42º 25.410´ S; 64º 07.867´ W	May 6, 2008	9.98±0.5	8.43±0.17	194±27	0.961±0.002	71.2±1.1	0.1	96.5	3.4	2.9	n.a.	0
Cordova Cove (CC)	CC08-1	45º 44.322´ S; 67º 22.695´ W	April 9, 2008	13.5±0.5	7.49±0.38	145±61	7.20±0.22	28.3±1.8	4.3	50.9	44.8	5002	0.3-0.4	1054
	CC08-2	45º 45.033´ S; 67º 22.486´ W	April 9, 2008	14.8±0.2	7.83±0.09	208±6	1.56 ±0.09	34.6±2.9	56.0	43.8	0.2	1669	1.1-0.9	758

AliHC: aliphatic hydrocarbons; **PAHs**: polyaromatic hydrocarbons; **Biod. index**: biodegradation indices (nC17/Pr-nC18/Ph); **dws**: dry weight sediment; n.a.: not applicable

Figure Legends

Fig. S1. Sampling locations at the Patagonian South Atlantic coast.

Fig. S2. Relation between EIHE and hydrocarbon exposure status, for pyrotag datasets from this and other published studies. Exposured status of the samples was categorized as oiled (black) and unoiled (grey). Analyses were performed with the STAMP software (Statistical Analysis of Metagenomic Profiles, http://kiwi.cs.dal.ca/Software/STAMP). A-Sediment samples from Patagonia (this study). B-Chronically-polluted Subantarctic sediments exposed to crude oil (19). Initial: original sediment sample. OR08.oil: sediment slurry after 20 days oil-exposure. OR08.oilnut: same as before with nutrients added. OR08.control: neither oil nor nutrient addition; C-Coastal mudflat sediments exposed to oil (7). CL and COL correspond to control and oiled conditions, respectively, while 2 and 21 correspond to 2 and 21 days of experiment, respectively; D-Brazilian mangrove sediments exposed to crude oil (11). Each condition includes the average value of two biological replicates. Control: no oil addition, 23 days after the onset of the experiment, 2%: same as above but exposed to 2% oil, 5% same as above but exposed to 5% oil; E-Beach sands impacted by the Deepwater Horizon oil spill (35). Samples were further categorized as “clean” and “oiled” according to visual contamination levels described in (35). Clean: visual level 0, oiled: visual levels 1-3. F- Seawater microcosms (Bagi A., unpublished, SRA061588). Samples were categorized as “arctic” and “temp” according to the seawater of origin (arctic or temperate), and “15ºC”, “8ºC”, “4ºC” and “0.5ºC” according to the temperature of incubation. G- Beach sands impacted by the Deepwater Horizon oil spill, discriminated by sampling event as well as oil status. “PB1” and “PB2” correspond to sampling events in Pensecola Beach, FL, 30 and 60 days after the spill, respectively. SGI: control site not impacted by the spill (Saint George Island, FL). Letters above the bars correspond to results from multiple comparisons following Kruskal-Wallis non parametric median test. The category “clean-PB2” contained only two datasets and therefore it was not included in statistical analysis.

Fig. S3. Detail of the relative contribution of the different genera accounting for the EIHE in this and other published studies, in oiled (black) and unoiled (grey) samples. A-Sediment samples from Patagonia (this study); B-Chronically-polluted Subantarctic sediments exposed to crude oil (19) ; **C**-Coastal mudflat sediments exposed to oil (7) D-Brazilian mangrove sediments exposed to crude oil (11); E-Beach sands impacted by the Deepwater Horizon oil spill (35); F- Seawater microcosms (Bagi A., unpublished, SRA061588).

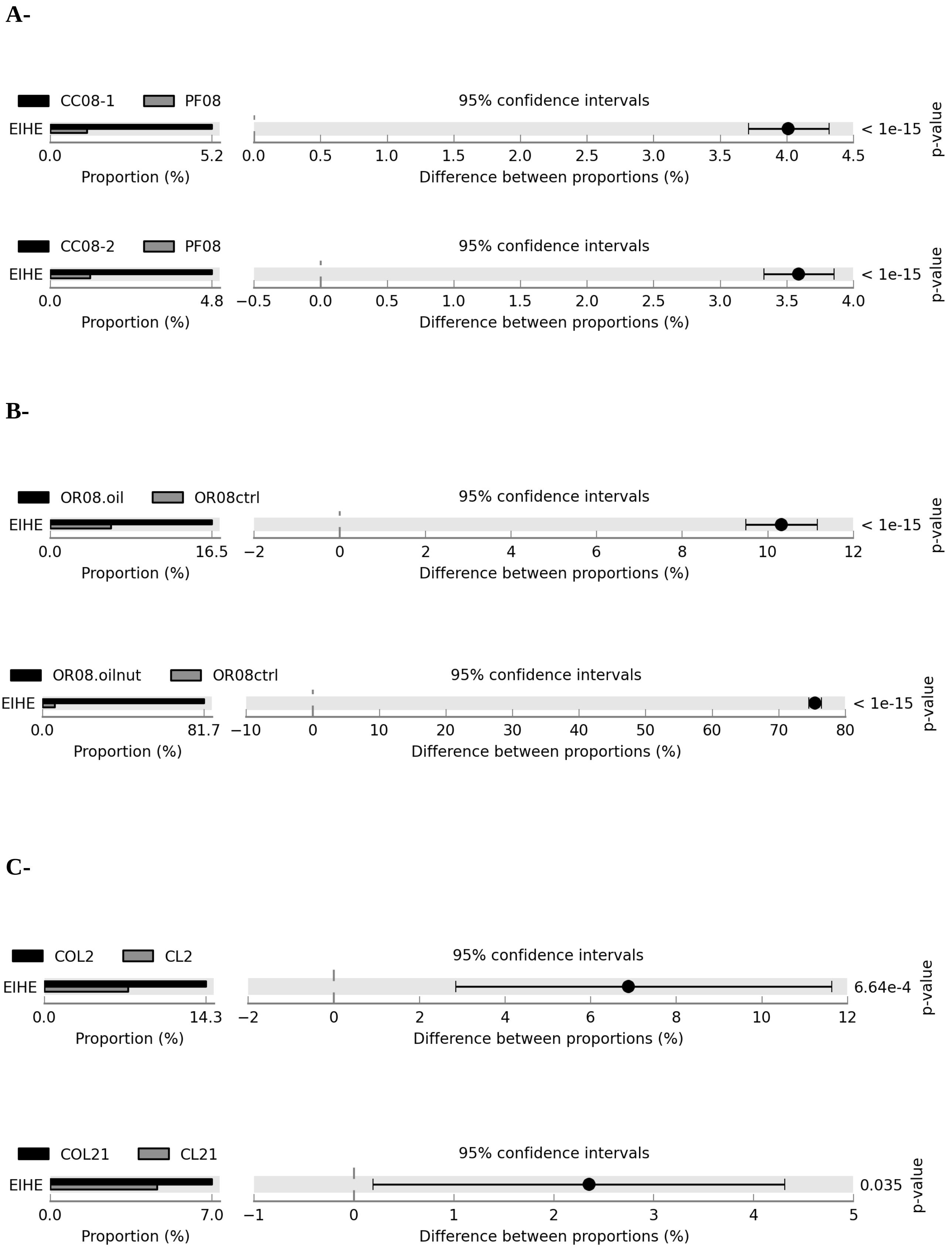

Lozada, Fig. S2

D-

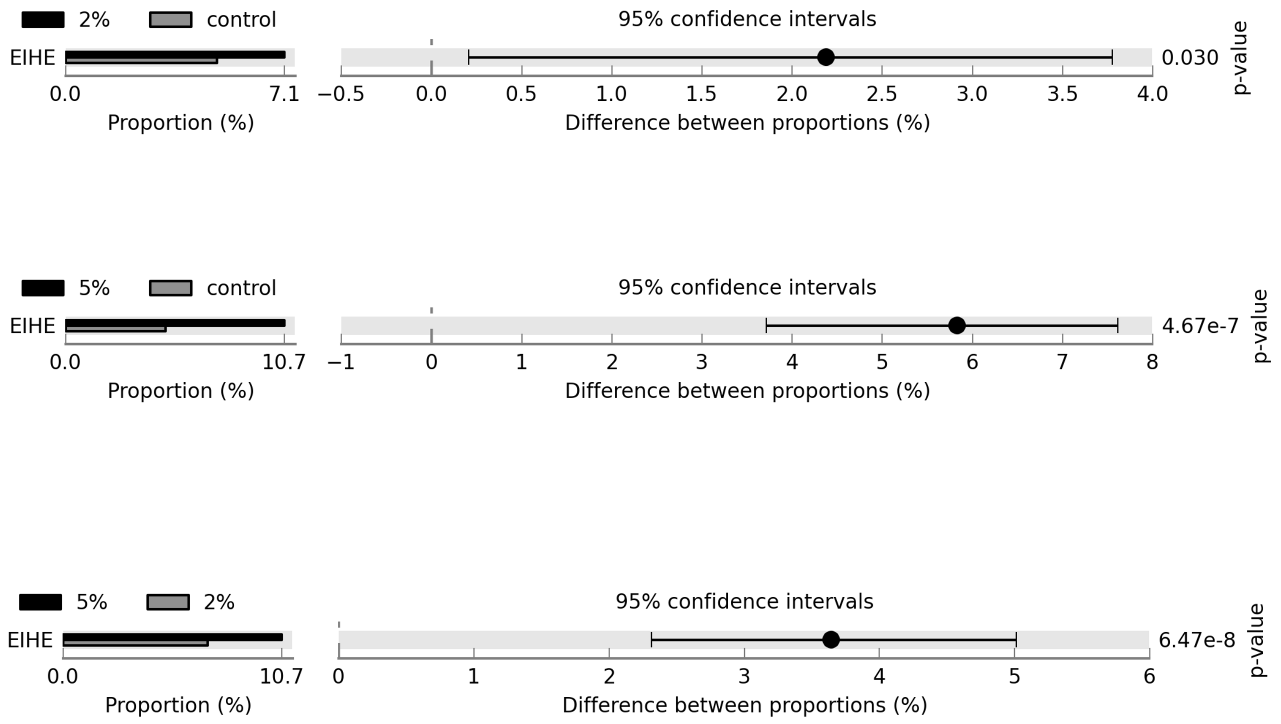

E-

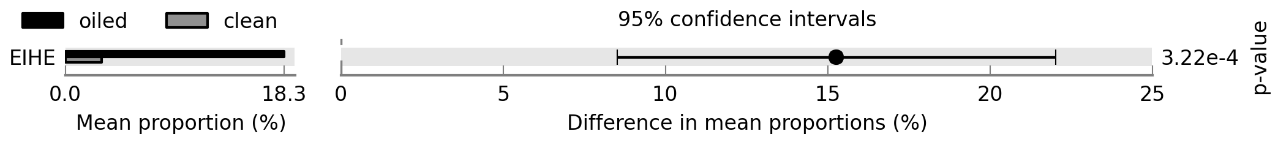

Lozada, Fig. S2(cont.)

F-

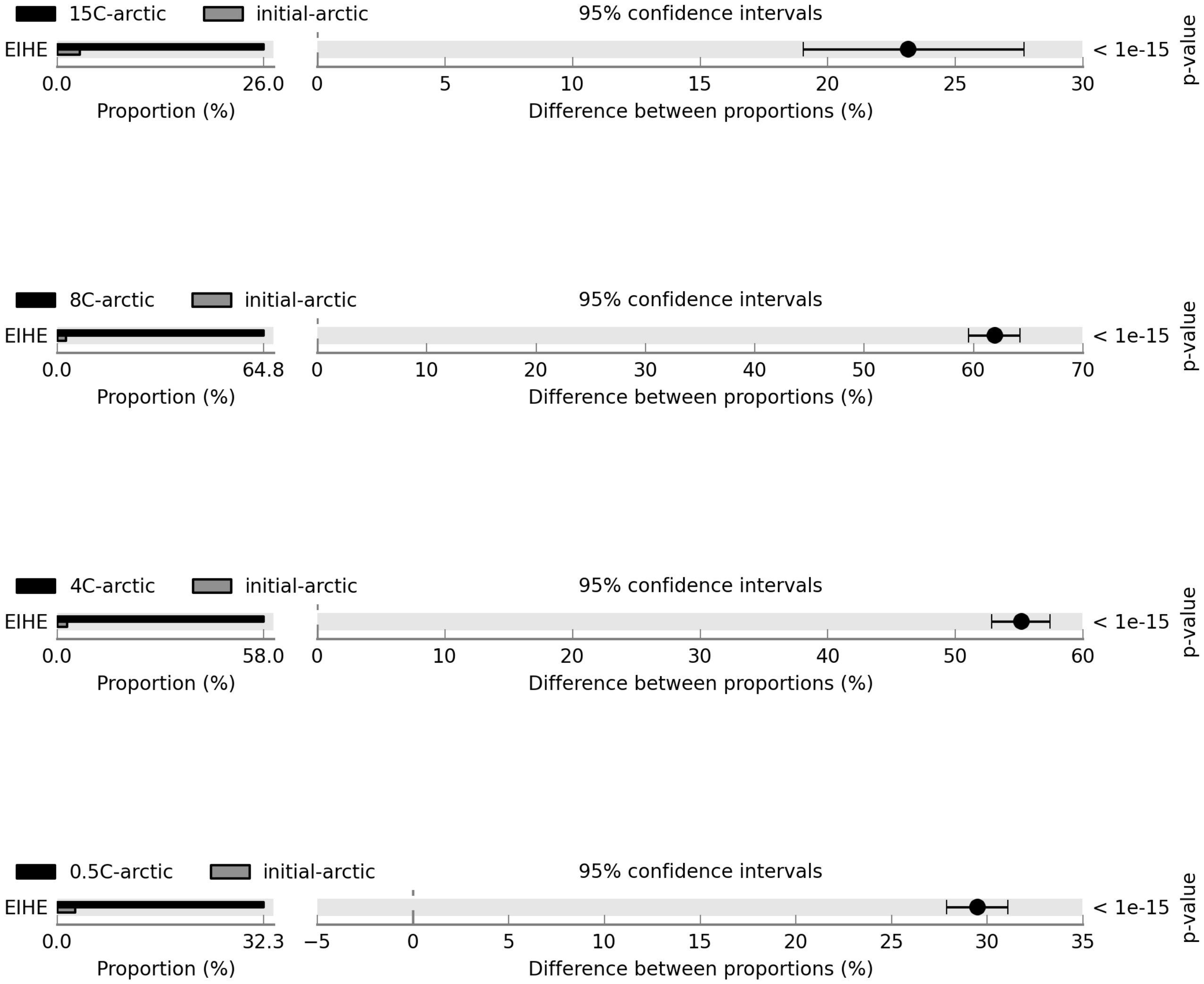

Lozada, Fig. S2 (cont.)

F-(cont.)

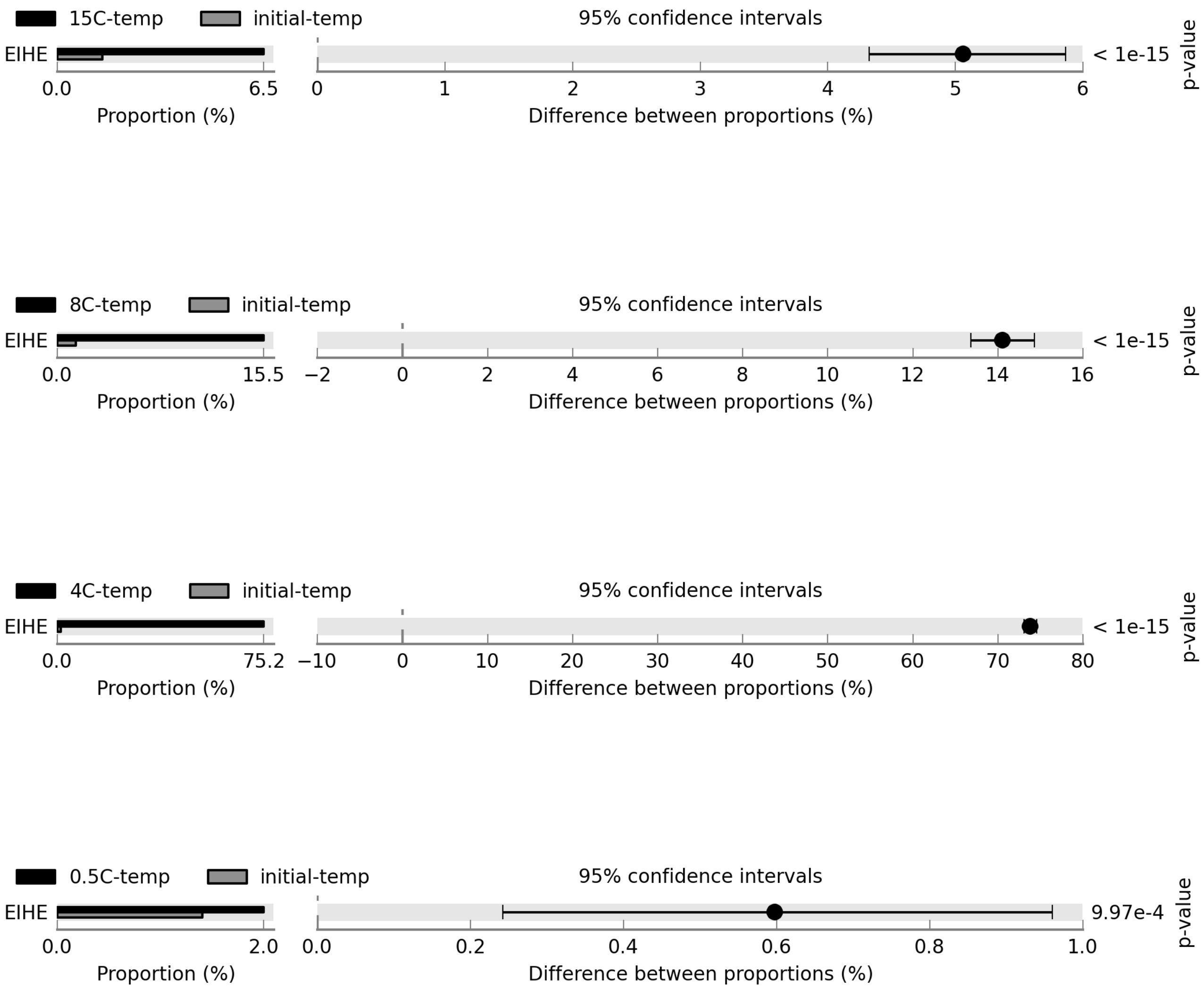

Lozada, Fig. S2 (cont.)

G-

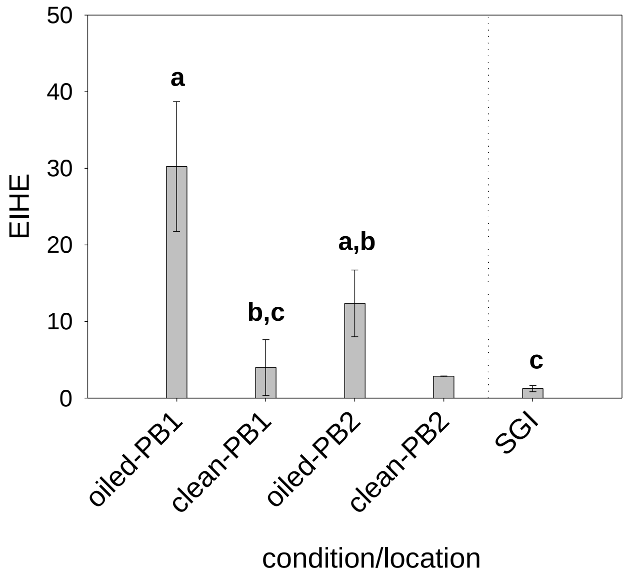

Lozada, Fig. S2 (cont.)

A-

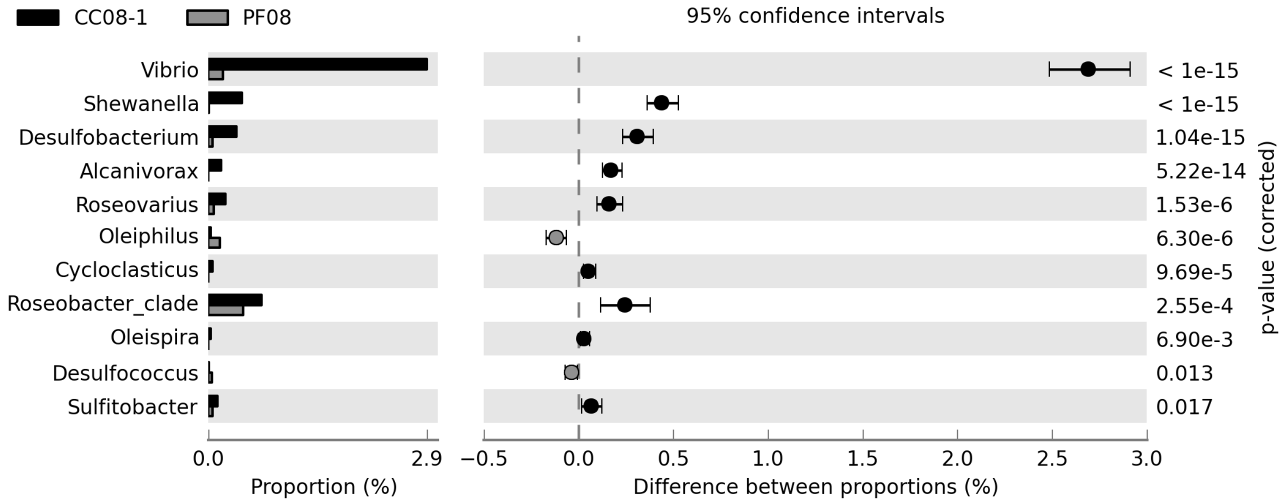

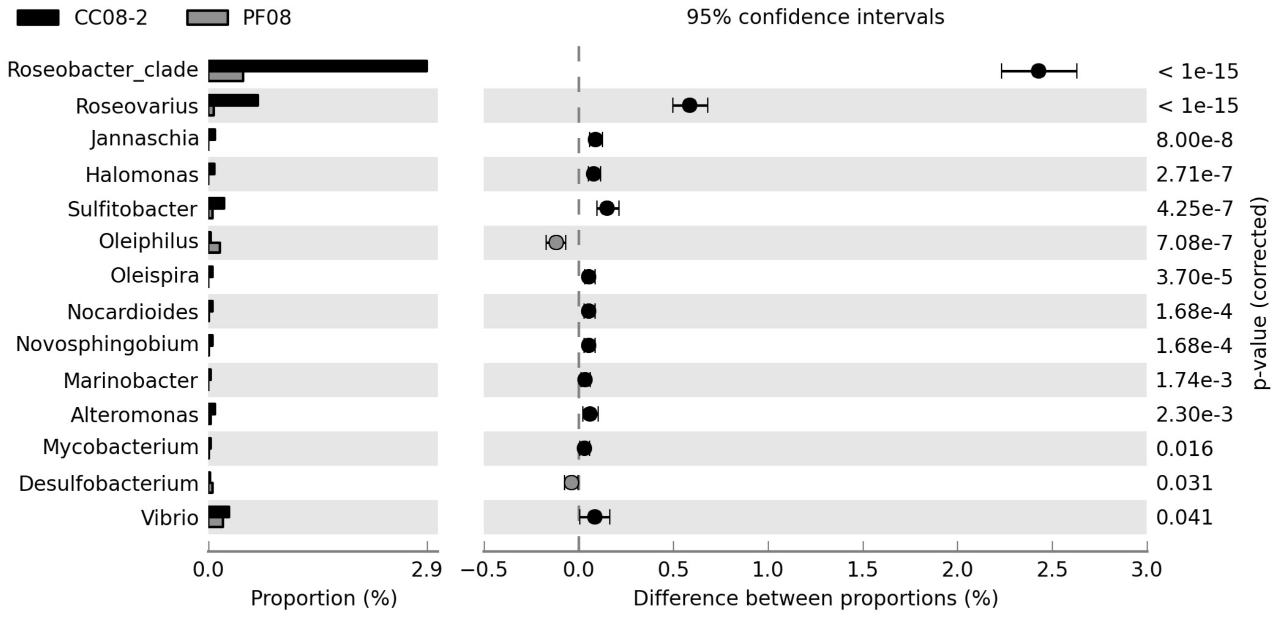

Lozada, Fig. S3

B-

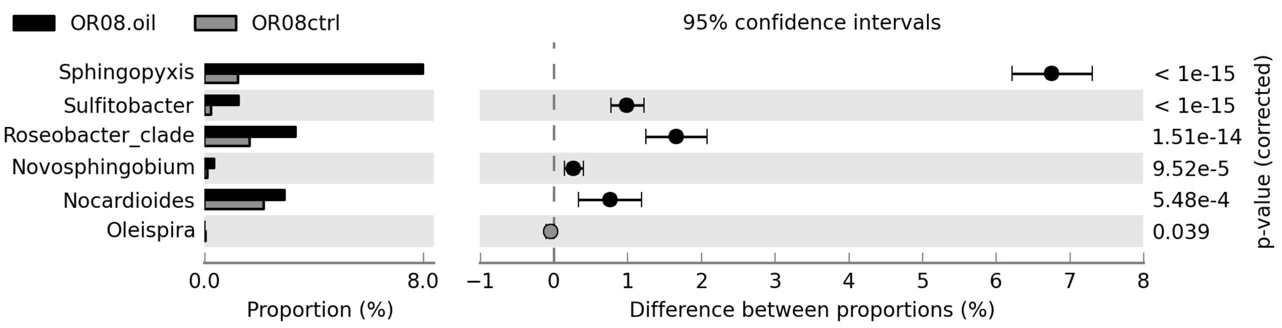

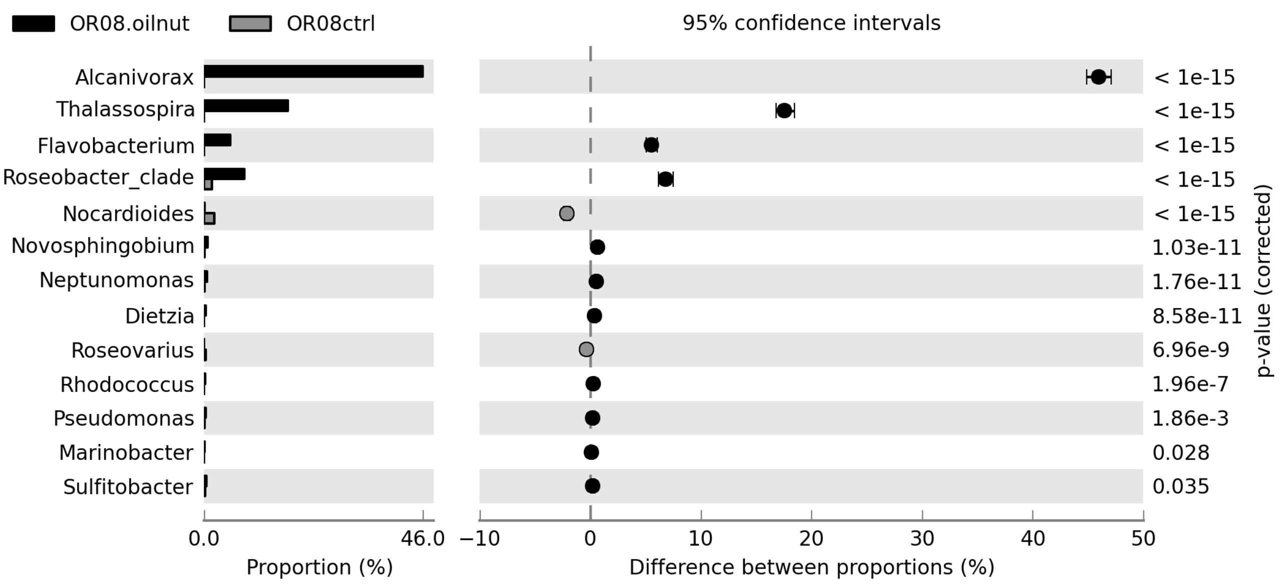

Lozada, Fig. S3 (cont.)

C-

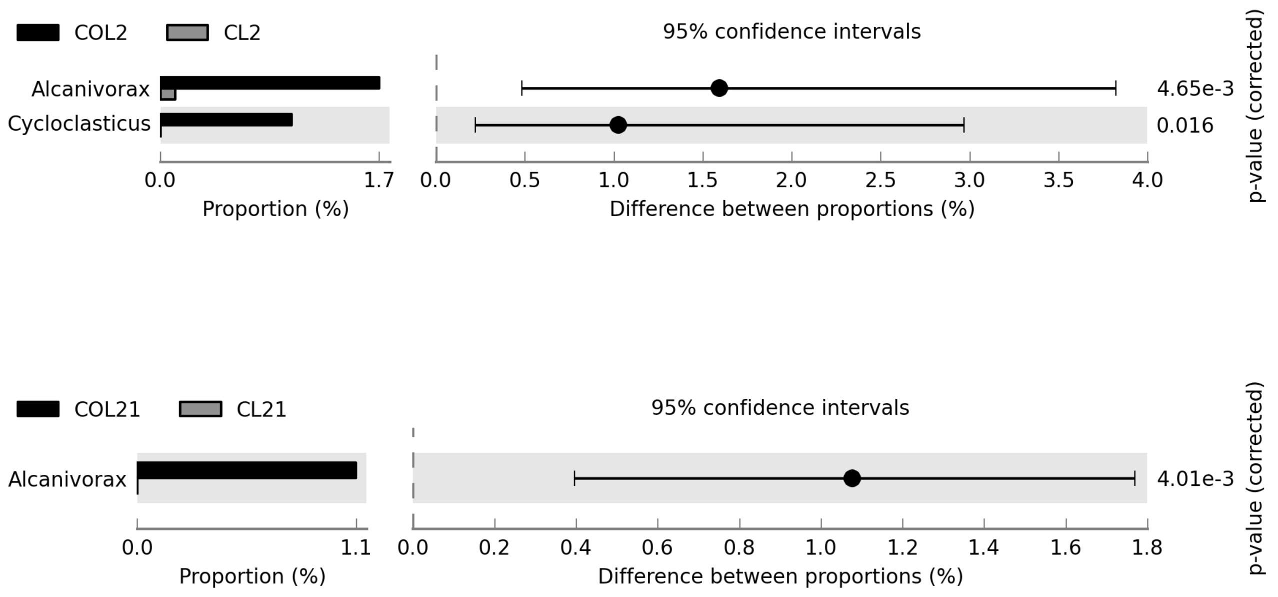

D-

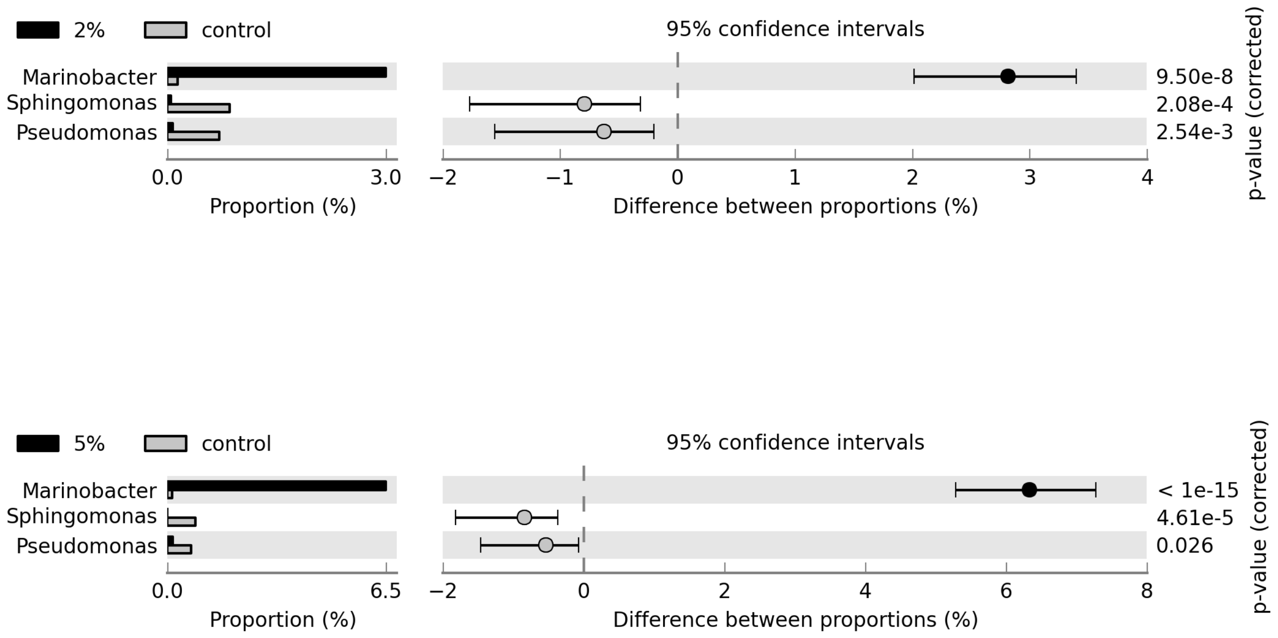

Lozada, Fig. S3 (cont.)

E-

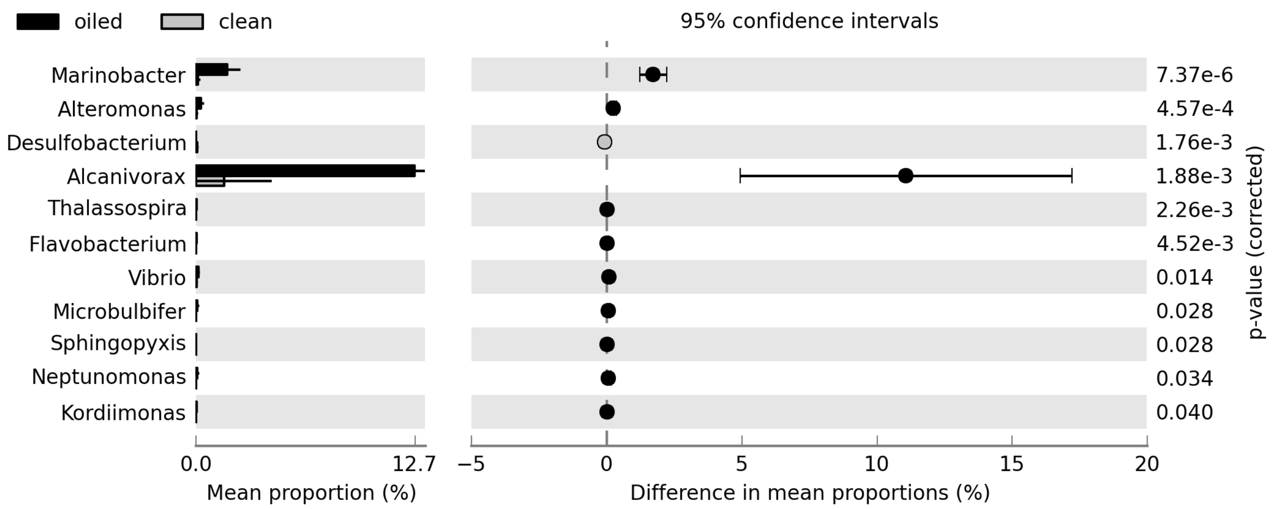

F-

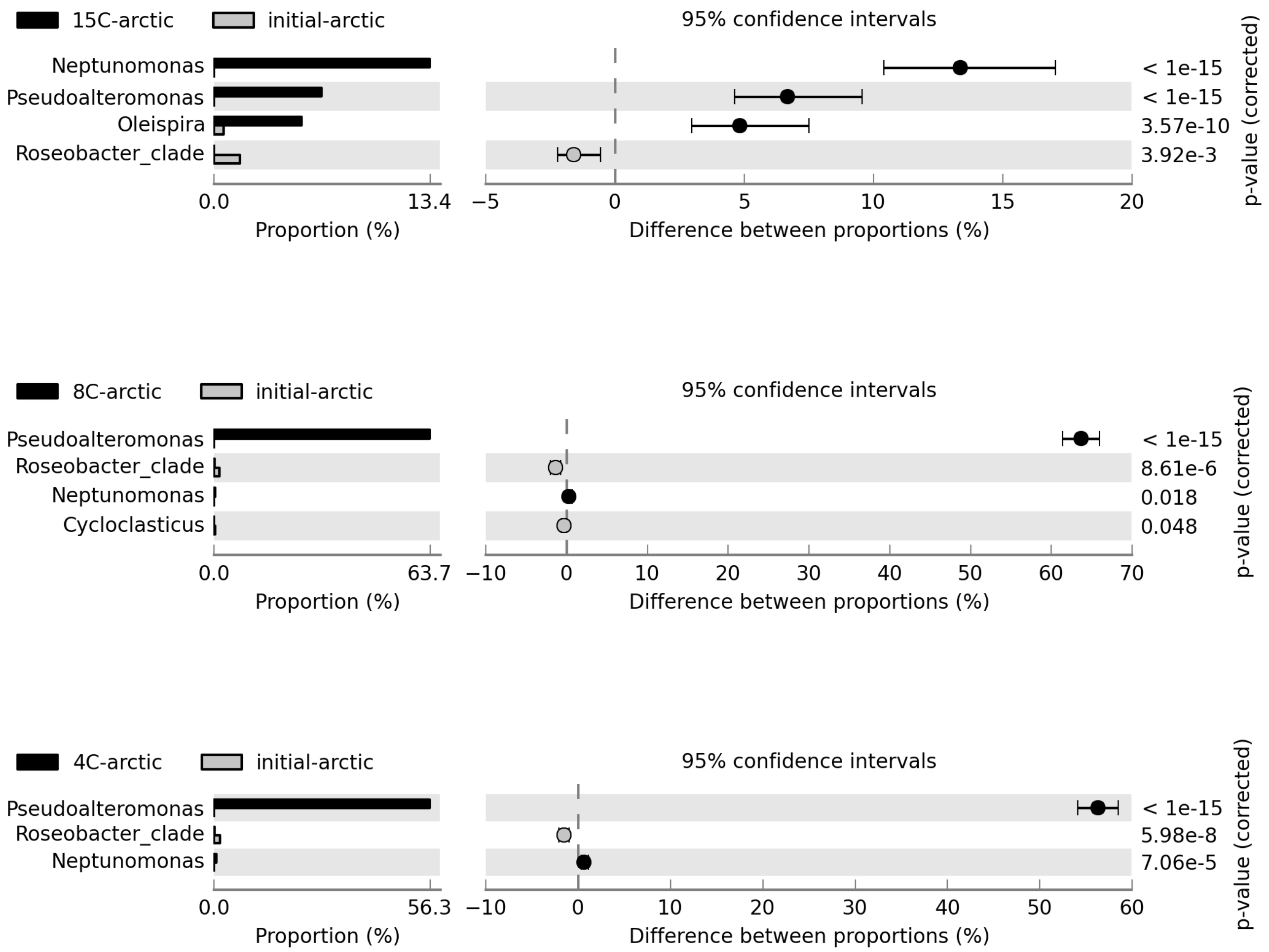

Lozada, Fig. S3(cont.)

F-(cont.)

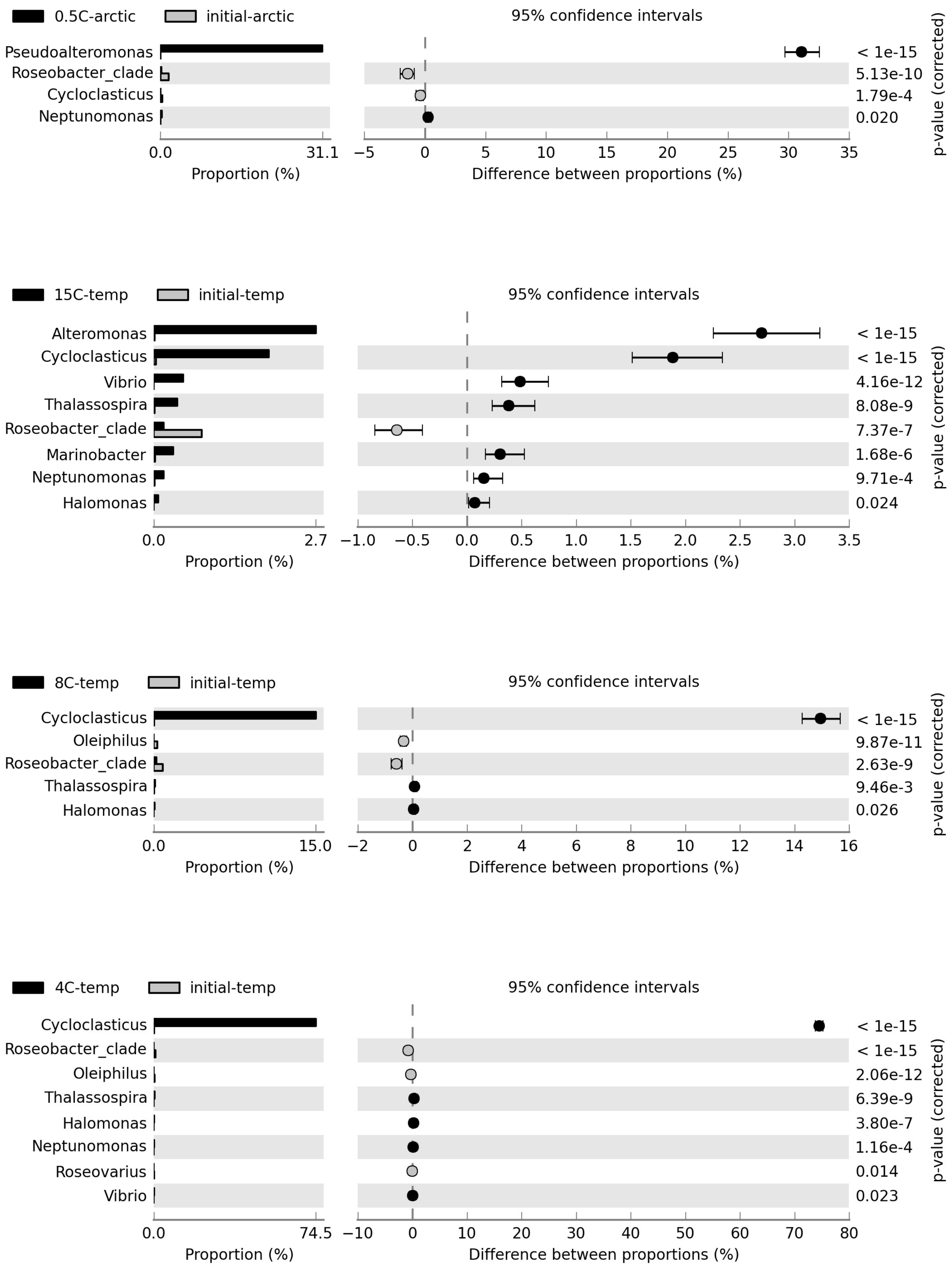

Lozada, Fig. S3(cont.)

F-(cont.)

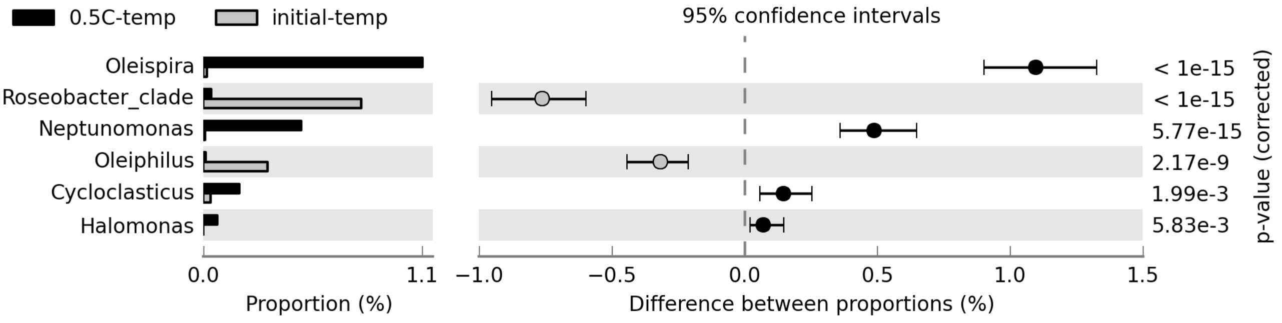

Lozada, Fig. S3(cont.)

APPENDIX

R script for calculating the Ecological Index of Hydrocarbon Exposure from mothur taxonomy summary file.

```
#     ECOLOGICAL INDEX OF HYDROCARBON EXPOSURE
#     DATE OF CREATION: SEPTEMBER 2012
#     AUTHOR: MARIANA LOZADA
#     DESCRIPTION: An ecological index of hydrocarbon exposure is calculated for each sequence dataset from each sample. This index is defined as the total sum of the relative abundances (as percent) of representative genera associated with hydrocarbon biodegradation (Lozada et al. 2012). The relative abundance of each genus is extracted as percentage, from the classification of sequences based on mothur (www.mothur.org). The script parses the mothur output table (the .tax.summary file, generated from the classify.seqs command), calculates percent abundances for the whole dataset, extracts the percent abundance of the selected genera, and performs the total sum of these in each sample (column).
#     INPUT
#     A dataframe (the .tax.summary file generated by the classify.seqs command of mothur)
#     OUTPUT/S
#     The output is a matrix. It is a space-delimited text file with the name of each sample and its corresponding index value. The table of the percent abundance of the selected genera extracted is also generated as an output.

input <- read.table(file="sediments.summary", header = TRUE, sep = "",
                  dec = ".", row.names = NULL,
                 as.is = TRUE) #we parse the table, it is tab-delimited and "." denotes decimal, the name of the file is just an example

str(input) #to see in R how the data look like
head(input) #same

#we will now calculate the percentages of each row with respect to bacteria. We must generate new columns (one per sample). The actual number of columns will depend on the number of samples in the dataset. After that we will attach them to the input dataframe

samples <- names(input)[6:dim(input)[2]] # object with the names of the samples taken from the names of the columns of the input table. The first 5 columns contain general information.

nsamples <-  length(samples) # number of samples

TablePercent <- matrix(NA, ncol = nsamples, nrow = nrow(input)) # matrix filled with NA and with columns equal to the number of samples and rows equal to the number of rows in the input to fill with results

colnames(TablePercent) <- paste(samples, "%", sep="") # name of the columns in the table of percentages

bacTot <- input[ input$taxon == "Bacteria", 6:ncol(input) ] # sample values of bacteria

for(i in 1:nsamples) TablePercent[,i] <- input[,5+i]/bacTot[1,i]*100 # percent abundance with respect to total bacteria
head(TablePercent)

write.table( cbind(input, TablePercent), "TotalPercentages.txt", sep=" " , dec=".", row.names=F, col.names=T)  # saves the table with % recently calculated attached to the original input data.

# Selecting data for bacteria associated with hydrocarbon biodegradation
```

```
BdBac <- c("Arthrobacter", "Dietzia", "Gordonia", "Microbacterium", "Micrococcus", "Mycobacterium", "Nocardia", "Nocardioides", "Prauserella", "Rhodococcus", "Streptomyces", "Terrabacter", "Cytophaga", "Flavobacterium", "Pedobacter", "Bacillus", "Paenibacillus", "Planomicrobium", "Kordiimonas", "Novosphingobium", "Ochrobactrum", "Sphingobium", "Sphingomonas", "Sphingopyxis", "Roseobacter_clade", "Roseovarius", "Jannaschia", "Silicibacter", "Sulfitobacter", "Thalassospira", "Tranquillimonas", "Tropicibacter", "Tropicimonas", "Acidovorax", "Alcaligenes", "Burkholderia", "Comamonas", "Delftia", "Polaromonas", "Ralstonia", "Desulfatibacillum", "Desulfatiferula", "Desulfobacterium", "Desulfococcus", "Desulfoglaeba", "Desulfothermus", "Alcanivorax", "Alkanindiges", "Cycloclasticus", "Oleibacter", "Oleiphilus", "Oleispira", "Thalassolituus", "Acinetobacter", "Alteromonas", "Halomonas", "Marinobacter", "Microbulbifer", "Neptunomonas", "Pseudoalteromonas", "Pseudomonas", "Shewanella", "Vibrio")

idx <- unlist(sapply(BdBac, function(x) which(input$taxon == x))) # index for selecting the hydrocarbon biodegradation bacteria data

write.table ( cbind(input[idx,], TablePercent[idx, ]) , file = "PercentBiodeg.txt", sep=" " , dec=".", row.names=F, col.names=T) # save it as a text table

indices <- colSums (TablePercent[idx, ]) # index: sum of % by sample of hydrocarbon biodegradation bacteria data
length(indices)
indices

write.table(indices, file = "index.txt", sep=" " , dec=".", row.names=T, col.names=F) #saves the index, this is the last output

#END
```

# ALTERNATIVE SCRIPT FOR PARSING MULTIPLE, ONE SAMPLE FILES

```
#First we have to set the working directory where the summary files are. Important note: these files must be altogether in the same folder, with no other files than these in the folder, in order not to have conflict with the generation of the index from the directory pathway.

setwd("C:/…/summary files")#example path

dir() #just to check that the folder is correct and no other files are there

files <- dir() #creates the object “files” corresponding to the name of the summary files in the directory

index <- data.frame(value = rep (NA, length=length(files)), sample=NA) #Object "index" is a two-column dataframe, the first one corresponding to the index value and the second to the name of the summary file (each one corresponding to one sample)

 for(i in 1:length(files)) {

      input<-read.table(file=files[i],
           header = TRUE, sep = "",
           dec = ".", row.names = NULL,
           as.is = TRUE)
               # parses the summary files one by one

tablepercent <- matrix (NA, ncol = 1, nrow = nrow(input)) # build the matrix corresponding to percentages

bac_tot <- input[input$taxon == "Bacteria", "total"] # total value for bacteria
```

```
percent <-  input[,5]/bac_tot*100  #percent values defined for all rows in the input table, and the columna Lumber 5 which corresponds to the number of sequences in the sample

tablepercent <- data.frame(input,percent) #write the table as text

percentbiodeg <- tablepercent[tablepercent$taxon
=="Arthrobacter"|tablepercent$taxon =="Dietzia"|tablepercent$taxon
=="Gordonia"|tablepercent$taxon =="Microbacterium"|tablepercent$taxon
=="Micrococcus"|tablepercent$taxon =="Mycobacterium"|tablepercent$taxon
=="Nocardia"|tablepercent$taxon =="Nocardioides"|tablepercent$taxon
=="Prauserella"|tablepercent$taxon =="Rhodococcus"|tablepercent$taxon
=="Streptomyces"|tablepercent$taxon =="Terrabacter"|tablepercent$taxon
=="Cytophaga"|tablepercent$taxon =="Flavobacterium"|tablepercent$taxon
=="Pedobacter"|tablepercent$taxon =="Bacillus"|tablepercent$taxon
=="Paenibacillus"|tablepercent$taxon =="Planomicrobium"|tablepercent$taxon
=="Kordiimonas"|tablepercent$taxon =="Novosphingobium"|tablepercent$taxon
=="Ochrobactrum"|tablepercent$taxon =="Sphingobium"|tablepercent$taxon
=="Sphingomonas"|tablepercent$taxon =="Sphingopyxis"|tablepercent$taxon
=="Roseobacter_clade"|tablepercent$taxon =="Roseovarius"|tablepercent$taxon
=="Jannaschia"|tablepercent$taxon =="Silicibacter"|tablepercent$taxon
=="Sulfitobacter"|tablepercent$taxon =="Thalassospira"|tablepercent$taxon
=="Tranquillimonas"|tablepercent$taxon =="Tropicibacter"|tablepercent$taxon
=="Tropicimonas"|tablepercent$taxon =="Acidovorax"|tablepercent$taxon
=="Alcaligenes"|tablepercent$taxon =="Burkholderia"|tablepercent$taxon
=="Comamonas"|tablepercent$taxon =="Delftia"|tablepercent$taxon
=="Polaromonas"|tablepercent$taxon =="Ralstonia"|tablepercent$taxon
=="Desulfatibacillum"|tablepercent$taxon =="Desulfatiferula"|tablepercent$taxon
=="Desulfobacterium"|tablepercent$taxon =="Desulfococcus"|tablepercent$taxon
=="Desulfoglaeba"|tablepercent$taxon =="Desulfothermus"|tablepercent$taxon
=="Alcanivorax"|tablepercent$taxon =="Alkanindiges"|tablepercent$taxon
=="Cycloclasticus"|tablepercent$taxon =="Oleibacter"|tablepercent$taxon
=="Oleiphilus"|tablepercent$taxon =="Oleispira"|tablepercent$taxon
=="Thalassolituus"|tablepercent$taxon =="Acinetobacter"|tablepercent$taxon
=="Alteromonas"|tablepercent$taxon =="Halomonas"|tablepercent$taxon
=="Marinobacter"|tablepercent$taxon =="Microbulbifer"|tablepercent$taxon
=="Neptunomonas"|tablepercent$taxon =="Pseudoalteromonas"|tablepercent$taxon
=="Pseudomonas"|tablepercent$taxon =="Shewanella"|tablepercent$taxon
=="Vibrio",] #selecting bacteria associated with biodegradation

O <- paste (files[i],"percentbiodeg", sep="-")# file name of each table of biodegradation associated genera which will be written next(see below)

write.table (percentbiodeg, file = O, append = FALSE, quote = TRUE, sep = ",",
            eol = "\n", na = "NA", dec = ".", row.names = FALSE,
            col.names = TRUE, qmethod = c("escape", "double"),
            fileEncoding = "") #writes the table as text

index[i,1] <-(sum (percentbiodeg [,6])) #calculates the index for each file
index[i,2] <- files[i] #name of the file (sample) in the second column
}
index

write.table( index,file = "index.txt", dec = ".", sep="," , row.names = TRUE,
                  col.names = FALSE) #index table for multiple samples, each corresponding to an individual summary file
```

References

1. Abed, R. M. M., N. Musat, F. Musat, and M. Mußmann. 2011. Structure of microbial communities and hydrocarbon-dependent sulfate reduction in the anoxic layer of a polluted microbial mat. Mar Pollut Bull. 62**:**539-546.
2. Barabas, G., G. Vargha, I. M. Szabo, A. Penyige, S. Damjanovich, J. Szollosi, J. Matko, T. Hirano, A. Matyus, and I. Szabo. 2001. N-Alkane uptake and utilisation by *Streptomyces* strains. Antonie Van Leeuwenhoek. 79**:**269-76.
3. Bogan, B. W., W. R. Sullivan, K. J. Kayser, K. Derr, H. C. Aldrich, and J. R. Paterek. 2003. *Alkanindiges illinoisensis* gen. nov., sp. nov., an obligately hydrocarbonoclastic, aerobic squalane-degrading bacterium isolated from oilfield soils. Int J Syst Evol Microbiol. 53**:**1389-1395.
4. Brito, E. M. S., R. Guyoneaud, M. Goñi-Urriza, A. Ranchou-Peyruse, A. Verbaere, M. A. C. Crapez, J. C. A. Wasserman, and R. Duran. 2006. Characterization of hydrocarbonoclastic bacterial communities from mangrove sediments in Guanabara Bay, Brazil. Res Microbiol. 157**:**752-762.
5. Buchan, A., and J. M. González. 2010. Roseobacter. Handbook of Hydrocarbon and Lipid Microbiology, p. 1335-1343. *In* K. N. Timmis (ed.). Springer Berlin Heidelberg.
6. Cerniglia, C. E. 1992. Biodegradation of polycyclic aromatic hydrocarbons. Biodegradation. 3**:**351-368.
7. Coulon, F., P.-M. Chronopoulou, A. Fahy, S. Paissé, M. Goñi-Urriza, L. Peperzak, L. Acuña Alvarez, B. A. McKew, C. P. D. Brussaard, G. J. C. Underwood, K. N. Timmis, R. Duran, and T. J. McGenity. 2012. Hydrocarbon biodegradation in coastal mudflats: the central role of dynamic tidal biofilms dominated by aerobic hydrocarbonoclastic bacteria and diatoms. Appl Environ Microbiol. doi:10.1128/aem.00072-12.
8. Cravo-Laureau, C., C. Labat, C. Joulian, R. Matheron, and A. Hirschler-Rea. 2007. *Desulfatiferula olefinivorans* gen. nov., sp. nov., a long-chain n-alkene-degrading, sulfate-reducing bacterium. Int J Syst Evol Microbiol. 57**:**2699-702.
9. Cravo-Laureau, C., R. Matheron, C. Joulian, J. L. Cayol, and A. Hirschler-Rea. 2004. *Desulfatibacillum alkenivorans* sp. nov., a novel n-alkene-degrading, sulfate-reducing bacterium, and emended description of the genus Desulfatibacillum. Int J Syst Evol Microbiol. 54**:**1639-42.
10. Davidova, I. A., K. E. Duncan, O. K. Choi, and J. M. Suflita. 2006. *Desulfoglaeba alkanexedens* gen. nov., sp. nov., an n-alkane-degrading, sulfate-reducing bacterium. Int J Syst Evol Microbiol. 56**:**2737-42.
11. Dos Santos, H. F., J. C. Cury, F. v. L. Do Carmo, A. L. Dos Santos, J. Tiedje, J. D. van Elsas, A. S. Rosado, and R. S. Peixoto. 2011. Mangrove bacterial diversity and the impact of oil contamination revealed by pyrosequencing: bacterial proxies for oil pollution. PLoS One. 6:e16943.
12. Dyksterhouse, S. E., J. P. Gray, R. P. Herwig, J. C. Lara, and J. T. Staley. 1995. *Cycloclasticus pugetii* gen. nov., sp. nov., an aromatic hydrocarbon-degrading bacterium from marine sediments. Int J Syst Bacteriol.. 45**:**116-123.
13. Engelhardt, M. A., K. Daly, R. P. Swannell, and I. M. Head. 2001. Isolation and characterization of a novel hydrocarbon-degrading, Gram-positive bacterium, isolated from intertidal beach sediment, and description of *Planococcus alkanoclasticus* sp. nov. J Appl Microbiol. 90**:**237-47.
14. Feitkenhauer, H., R. Muller, and H. Markl. 2003. Degradation of polycyclic aromatic hydrocarbons and long chain alkanes at 60-70 degrees C by *Thermus* and *Bacillus* spp [corrected]. Biodegradation. 14**:**367-72.
15. Garcia-Valdes, E., E. Cozar, R. Rotger, J. Lalucat, and J. Ursing. 1988. New naphthalene-degrading marine *Pseudomonas* strains. Appl Environ Microbiol. 54**:**2478-2485.

16. Gauthier, M., B. Lafay, R. Christen, L. Fernandez, M. Acquaviva, P. Bonin, and J. Bertrand. 1992. *Marinobacter hydrocarbonoclasticus* gen. nov., sp. nov., a new, extremely halotolerant, hydrocarbon-degrading marine bacterium. Int J Syst Bacteriol. 42:568-576.
17. Gentile, G., V. Bonasera, C. Amico, L. Giuliano, and M. Yakimov. 2003. *Shewanella* sp. GA-22, a psychrophilic hydrocarbonoclastic antarctic bacterium producing polyunsaturated fatty acids. J Appl Microbiol. 95**:**1124-1133.
18. Golyshin, P. N., T. N. Chernikova, W. R. Abraham, L. Heinrich, K. N. Timmis, and M. M. Yakimov. 2002. Oleiphilaceae fam. nov., to include *Oleiphilus messinensis* gen. nov., sp. nov., a novel marine bacterium that obligately utilizes hydrocarbons. Int J Syst Evol Microbiol. 52**:**901-911.
19. Guibert, L. M., C. L. Loviso, M. S. Marcos, M. G. Commendatore, H. M. Dionisi, and M. Lozada. 2012. Alkane biodegradation genes from chronically polluted Subantarctic coastal sediments and their shifts in response to oil exposure. Microbial Ecology. 74:20-31.
20. Hamamura, N., and D. J. Arp. 2000. Isolation and characterization of alkane-utilizing *Nocardioides* sp. strain CF8. FEMS Microbiol Lett. 186:21-26.
21. Harms, G., K. Zengler, R. Rabus, F. Aeckersberg, D. Minz, R. Rosselló-Mora, and F. Widdel. 1999. Anaerobic oxidation of o-xylene, m-xylene, and homologous alkylbenzenes by new types of sulfate-reducing bacteria. Appl Environ Microbiol. 65:999-1004.
22. Harwati, T. U., Y. Kasai, Y. Kodama, D. Susilaningsih, and K. Watanabe. 2008. *Tranquillimonas alkanivorans* gen. nov., sp. nov., an alkane-degrading bacterium isolated from Semarang Port in Indonesia. Int J Syst Evol Microbiol. 58:2118-21.
23. Harwati, T. U., Y. Kasai, Y. Kodama, D. Susilaningsih, and K. Watanabe. 2009. *Tropicibacter naphthalenivorans* gen. nov., sp. nov., a polycyclic aromatic hydrocarbon-degrading bacterium isolated from Semarang Port in Indonesia. Int J Syst Evol Microbiol. 59:392-6.
24. Harwati, T. U., Y. Kasai, Y. Kodama, D. Susilaningsih, and K. Watanabe. 2009. *Tropicimonas isoalkanivorans* gen. nov., sp. nov., a branched-alkane-degrading bacterium isolated from Semarang Port in Indonesia. Int J Syst Evol Microbiol. 59:388-91.
25. Hedlund, B. P., A. D. Geiselbrecht, T. J. Bair, and J. T. Staley. 1999. Polycyclic aromatic hydrocarbon degradation by a new marine bacterium, *Neptunomonas naphthovorans* gen. nov., sp. nov. Appl Environ Microbiol. 65:251-259.
26. Hedlund, B. P., and J. T. Staley. 2006. Isolation and characterization of *Pseudoalteromonas* strains with divergent polycyclic aromatic hydrocarbon catabolic properties. Environ Microbiol. 8:178-182.
27. Hedlund, B. P., and J. T. Staley. 2001. *Vibrio cyclotrophicus* sp. nov., a polycyclic aromatic hydrocarbon (PAH)-degrading marine bacterium. Int J Syst Evol Microbiol. 51:61-66.
28. Iida, T., Y. Mukouzaka, K. Nakamura, and T. Kudo. 2002. Plasmid-borne genes code for an angular dioxygenase involved in dibenzofuran degradation by *Terrabacter* sp. strain YK3. Appl Environ Microbiol. 68:3716-23.
29. Ilori, M. O., D. Amund, and G. K. Robinson. 2000. Ultrastructure of two oil-degrading bacteria isolated from the tropical soil environment. Folia Microbiol (Praha). 45:259-62.
30. Jeon, C. O., W. Park, W. C. Ghiorse, and E. L. Madsen. 2004. *Polaromonas naphthalenivorans* sp. nov., a naphthalene-degrading bacterium from naphthalene-contaminated sediment. Int J Syst Evol Microbiol. 54:93-7.
31. Jin, H. M., H. Jeong, E. J. Moon, R. K. Math, K. Lee, H. J. Kim, C. O. Jeon, T. K. Oh, and J. F. Kim. 2011. Complete genome sequence of the polycyclic aromatic hydrocarbon-degrading bacterium *Alteromonas* sp. Strain SN2. J Bacteriol. 193:4292-4293.
32. Kertesz, M., and A. Kawasaki. 2010. Hydrocarbon-degrading sphingomonads: *Sphingomonas*, *Sphingobium*, *Novosphingobium*, and *Sphingopyxis*. Handbook of Hydrocarbon and Lipid Microbiology. Springer-Verlag Berlin:1693-1705.
33. Khomiakova, D. V., I. V. Botvinko, and A. I. Netrusov. 2003. [Isolation of hydrocarbon-oxidizing psychrophilic bacteria from oil-polluted soils]. Prikl Biokhim Mikrobiol. 39:661-4.

34. Kodama, Y., L. I. Stiknowati, A. Ueki, K. Ueki, and K. Watanabe. 2008. *Thalassospira tepidiphila* sp. nov., a polycyclic aromatic hydrocarbon-degrading bacterium isolated from seawater. Int J Syst Evol Microbiol. 58:711-5.
35. Kostka, J. E., O. Prakash, W. A. Overholt, S. J. Green, G. Freyer, A. Canion, J. Delgardio, N. Norton, T. C. Hazen, and M. Huettel. 2012. Hydrocarbon-degrading bacteria and the bacterial community response in Gulf of Mexico beach sands impacted by the Deepwater Horizon oil spill. Appl Environ Microbiol. 77:7962-7974.
36. Kuever, J., F. Rainey, and F. Widdel. 2005. *Desulfothermus* gen. nov. Bergey's Manual of Systematic Bacteriology:955-956.
37. Kwon, K., and S. Kim. 2010. Marine, hydrocarbon-degrading *Alphaproteobacteria*. Handbook of Hydrocarbon and Lipid Microbiology. 19:1707-1714.
38. Kwon, K. K., H. S. Lee, S. H. Yang, and S. J. Kim. 2005. *Kordiimonas gwangyangensis* gen. nov., sp. nov., a marine bacterium isolated from marine sediments that forms a distinct phyletic lineage (Kordiimonadales ord. nov.) in the 'Alphaproteobacteria'. Int J Syst Evol Microbiol. 55:2033-7.
39. Marcos, M. S., M. Lozada, W. D. Di Marzio, and H. M. Dionisi. 2012. Abundance, dynamics, and biogeographic distribution of seven polycyclic aromatic hydrocarbon dioxygenase gene variants in coastal sediments of Patagonia. Appl Environ Microbiol. 78:1589-1592.
40. Margesin, R., C. Sproer, P. Schumann, and F. Schinner. 2003. *Pedobacter cryoconitis* sp. nov., a facultative psychrophile from alpine glacier cryoconite. Int J Syst Evol Microbiol. 53:1291-6.
41. Meyer, S., R. Moser, A. Neef, U. Stahl, and P. Kampfer. 1999. Differential detection of key enzymes of polyaromatic-hydrocarbon-degrading bacteria using PCR and gene probes. Microbiology. 145 (Pt 7):1731-41.
42. Mikolasch, A., E. Hammer, and F. Schauer. 2003. Synthesis of imidazol-2-yl amino acids by using cells from alkane-oxidizing bacteria. Appl Environ Microbiol. 69:1670-9.
43. Parales, R. 2010. 4 Hydrocarbon Degradation by Betaproteobacteria. Handbook of Hydrocarbon and Lipid Microbiology:1715-1724.
44. Parales, R. E., J. L. Ditty, and C. S. Harwood. 2000. Toluene-degrading bacteria are chemotactic towards the environmental pollutants benzene, toluene, and trichloroethylene. Appl Environ Microbiol. 66:4098-104.
45. Passeri, A., S. Lang, F. Wagner, and V. Wray. 1991. Marine biosurfactants, II. Production and characterization of an anionic trehalose tetraester from the marine bacterium Arthrobacter sp. EK 1. Z Naturforsch C. 46:204-9.
46. Peressutti, S. R., H. M. Alvarez, and O. H. Pucci. 2003. Dynamics of hydrocarbon-degrading bacteriocenosis of an experimental oil pollution in Patagonian soil. International Biodeterioration & Biodegradation. 52:21-30.
47. Schippers, A., K. Bosecker, C. Sproer, and P. Schumann. 2005. *Microbacterium oleivorans* sp. nov. and *Microbacterium hydrocarbonoxydans* sp. nov., novel crude-oil-degrading Gram-positive bacteria. Int J Syst Evol Microbiol. 55:655-60.
48. Smits, T. H., S. B. Balada, B. Witholt, and J. B. van Beilen. 2002. Functional analysis of alkane hydroxylases from gram-negative and gram-positive bacteria. J Bacteriol. 184:1733-42.
49. Sohn, J. H., K. K. Kwon, J. H. Kang, H. B. Jung, and S. J. Kim. 2004. Novosphingobium pentaromativorans sp. nov., a high-molecular-mass polycyclic aromatic hydrocarbon-degrading bacterium isolated from estuarine sediment. Int J Syst Evol Microbiol. 54:1483-7.
50. Stucki, G., and M. Alexander. 1987. Role of dissolution rate and solubility in biodegradation of aromatic compounds. Appl Environ Microbiol. 53:292-7.
51. Teramoto, M., M. Ohuchi, A. Hatmanti, Y. Darmayati, Y. Widyastuti, S. Harayama, and Y. Fukunaga. 2011. *Oleibacter marinus* gen. nov., sp. nov., a bacterium that degrades

petroleum aliphatic hydrocarbons in a tropical marine environment. Int J Syst Evol Microbiol 61:375-380.

52. Toledo, F. L., J. Gonzalez-Lopez, and C. Calvo. 2008. Production of bioemulsifier by Bacillus subtilis, *Alcaligenes faecalis* and *Enterobacter* species in liquid culture. Bioresource Technol. 99:8470-8475.

53. Wang, Y. N., H. Cai, C. Q. Chi, A. H. Lu, X. G. Lin, Z. F. Jiang, and X. L. Wu. 2007. *Halomonas shengliensis* sp. nov., a moderately halophilic, denitrifying, crude-oil-utilizing bacterium. Int J Syst Evol Microbiol 57:1222-1226.

54. Willumsen, P., U. Karlson, E. Stackebrandt, and R. M. Kroppenstedt. 2001. *Mycobacterium frederiksbergense* sp. nov., a novel polycyclic aromatic hydrocarbon-degrading *Mycobacterium* species. Int J Syst Evol Microbiol. 51:1715-22.

55. Xue, Y., X. Sun, P. Zhou, R. Liu, F. Liang, and Y. Ma. 2003. *Gordonia paraffinivorans* sp. nov., a hydrocarbon-degrading actinomycete isolated from an oil-producing well. Int J Syst Evol Microbiol. 53:1643-6.

56. Yakimov, M. M., L. Giuliano, R. Denaro, E. Crisafi, T. N. Chernikova, W. R. Abraham, H. Luensdorf, K. N. Timmis, and P. N. Golyshin. 2004. *Thalassolituus oleivorans* gen. nov., sp. nov., a novel marine bacterium that obligately utilizes hydrocarbons. Int J Syst Evol Microbiol. 54:141-148.

57. Yakimov, M. M., L. Giuliano, G. Gentile, E. Crisafi, T. N. Chernikova, W. R. Abraham, H. Lünsdorf, K. N. Timmis, and P. N. Golyshin. 2003. *Oleispira antarctica* gen. nov., sp. nov., a novel hydrocarbonoclastic marine bacterium isolated from Antarctic coastal sea water. Int J Syst Evol Microbiol. 53:779-785.

58. Yakimov, M. M., P. N. Golyshin, S. Lang, E. R. Moore, W. R. Abraham, H. Lunsdorf, and K. N. Timmis. 1998. *Alcanivorax borkumensis* gen. nov., sp. nov., a new, hydrocarbon-degrading and surfactant-producing marine bacterium. Int J Syst Bacteriol. 48:339-348.

59. Yumoto, I., A. Nakamura, H. Iwata, K. Kojima, K. Kusumoto, Y. Nodasaka, and H. Matsuyama. 2002. *Dietzia psychralcaliphila* sp. nov., a novel, facultatively psychrophilic alkaliphile that grows on hydrocarbons. Int J Syst Evol Microbiol. 52:85-90.
